# Hypertension in Rural India: The Contribution of Socioeconomic Position

**DOI:** 10.1161/JAHA.119.014486

**Published:** 2020-03-30

**Authors:** Amanda G. Thrift, Rathina Srinivasa Ragavan, Michaela A. Riddell, Rohina Joshi, K. R. Thankappan, Clara Chow, Brian Oldenburg, Ajay S. Mahal, Kartik Kalyanram, Kamakshi Kartik, Oduru Suresh, G. K. Mini, Jordan Ismail, Dilan Giguruwa Gamage, Aniqa Hasan, Velandai K. Srikanth, Nihal Thomas, Pallab K. Maulik, Rama K. Guggilla, Roger G. Evans

**Affiliations:** ^1^ School of Clinical Sciences at Monash Health Monash University Melbourne Australia; ^2^ The George Institute for Global Health University of New South Wales Australia; ^3^ Achutha Menon Centre for Health Science Studies Sree Chitra Tirunal Institute for Medical Sciences and Technology Trivandrum Kerala, India; ^4^ Department of Cardiology Westmead Hospital Sydney Australia; ^5^ Melbourne School of Population and Global Health University of Melbourne Carlton Australia; ^6^ School of Public Health and Preventative Medicine Monash University Melbourne Australia; ^7^ Nossal Institute for Global Health Melbourne School of Population and Global Health University of Melbourne Carlton Australia; ^8^ Rishi Valley Rural Health Centre Chittoor District Andhra Pradesh India; ^9^ Global Institute of Public Health Ananthapuri Hospitals and Research Institute Trivandrum Kerala India; ^10^ Peninsula Clinical School Central Clinical School Monash University Frankston Australia; ^11^ Department of Endocrinology, Diabetes and Metabolism Christian Medical College Vellore Tamil Nadu India; ^12^ George Institute for Global Health New Delhi India; ^13^ George Institute for Global Health–Oxford University Oxford United Kingdom; ^14^ Department of Population Medicine and Civilization Diseases Prevention Faculty of Medicine With the Division of Dentistry and Division of Medical Education in English Medical University of Bialystok Bialystok Poland; ^15^ Cardiovascular Disease Program Biomedicine Discovery Institute, and Department of Physiology Monash University Melbourne Australia

**Keywords:** education, lifestyle, low‐to‐middle income, risk factors, socioeconomic position, Epidemiology, Lifestyle, Risk Factors, High Blood Pressure, Hypertension

## Abstract

**Background:**

Various indicators of socioeconomic position (SEP) may have opposing effects on the risk of hypertension in disadvantaged settings. For example, high income may reflect sedentary employment, whereas greater education may promote healthy lifestyle choices. We assessed whether education modifies the association between income and hypertension in 3 regions of South India at different stages of epidemiological transition.

**Methods and Results:**

Using a cross‐sectional design, we randomly selected villages within each of rural Trivandrum, West Godavari, and Rishi Valley. Sampling was stratified by age group and sex. We measured blood pressure and anthropometry and administered a questionnaire to identify lifestyle factors and SEP, including education, literacy, and income. Logistic regression was used to assess associations between various components of SEP and hypertension, and interaction analyses were used to determine whether educational attainment modified the association between income and hypertension. Trivandrum, the region of highest SEP, had the greatest prevalence of hypertension, whereas Rishi Valley, the lowest SEP region, had the least. Overall, greater income was associated with greater risk of hypertension. In interaction analyses, there was no evidence that educational attainment modified the association between income and hypertension.

**Conclusions:**

Education is widely considered to ameliorate the risk of hypertension in high‐income countries. Why this effect is absent in rural India merits investigation.

**Clinical Perspective**

**What Is New?**

- We found that the risk of hypertension was positively associated with higher socioeconomic position (SEP) in rural India.
- We also found that modifiable risk factors, such as greater adiposity, may collectively mediate the increased risk of hypertension in individuals from higher socioeconomic backgrounds.

**What Are the Clinical Implications?**

- Health education and prevention strategies that are targeted at those who are at high risk of hypertension, such as retirees and older unemployed people, may provide an important means to reduce the emergence of hypertension in rural India.

## Introduction

Recently, the prevalence of hypertension in low‐ to middle‐income countries has been estimated to exceed that in high‐income countries (HICs).^1^ Socioeconomic position (SEP), generally measured using indicators such as education, income, and occupation,^2^ is strongly associated with the presence of hypertension in HICs,^3^ with individuals of higher SEP less likely to have hypertension than those of lower SEP.^4^ The association between SEP and hypertension in HICs may be attributable to a greater awareness of hypertension and associated risk factors in those with high SEP, and greater access and adherence to health care.^5^ However, in low‐ to middle‐income countries, there is some evidence that higher SEP may be associated with a greater risk of hypertension,^6^ although findings have been inconsistent.^3^, ^7^, ^8^ The association between elevated SEP and poor health in these settings may be driven by changes in behavior, such as excessive consumption of alcohol, excessive calorie intake, or the greater likelihood of sedentary employment, in higher SEP brackets.^9^, ^10^, ^11^

Education may be a critical factor potentially mitigating the negative impacts of economic development on hypertension by empowering individuals with the knowledge to improve their health.^4^, ^9^ To our knowledge, the relative effects of income and education on the risk of hypertension have not been investigated in disadvantaged settings. We tested the hypothesis that education mitigates the association between income and hypertension by determining the associations of educational attainment and income with hypertension, and the interaction between educational attainment and income, in 3 economically diverse regions of India. We also investigated the relationship between SEP and risk factors for hypertension.

## Methods

### Data Statement

To minimize the possibility of unintentionally sharing information that can be used to reidentify private information, a subset of the data generated for this study is available at the Monash University Bridges and can be accessed at DOI 10.26180/5e212eb30b4f4.

### Study Region

The 3 study regions have differing levels of educational attainment, income, and occupations. A rural region in the northern part of the District of Trivandrum (herein referred to as Trivandrum) in Kerala is the most socioeconomically advanced region, West Godavari District (herein referred to as Godavari) in Northern Andhra Pradesh is less advanced, and Rishi Valley region (Chittoor District) in Southern Andhra Pradesh is the least socioeconomically advanced (Data S1).^12^

### Study Design

**Figure 1:**
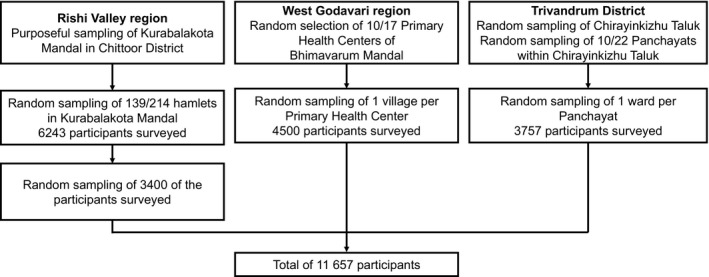
Flow diagram of participants in 3 rural regions in India, 2014 to 2015. The 8 Mandals in West Godavari were Palakoderu, Undrajavaram, Iragavaram, Mogalthur, Unguturu, Pentapadu, Penumantra, and Attili. Participation was as follows: Rishi Valley, 45%; Godavari, 99%; and Trivandrum, 77%.

Villages (clusters) were randomly selected within Rishi Valley, Godavari, and Trivandrum for inclusion. This involved dividing each of the 3 sites into primary sampling units (villages, wards, or hamlets) by computer‐generated random selection. In each primary sampling unit, a full list of residents was obtained and then individuals were sampled into 12 categories by age (18–24, 25–34, 35–44, 45–54, 55–64, and ≥65 years) and sex. In an effort to reduce bias, eligible participants were revisited when unavailable on the first or second visit to the village. Using this method, 11 657 participants were recruited between January 2014 and December 2015 (Figure 1).

Our sample size was based on outcomes for a cluster randomized controlled trial (registered with the Clinical Trials Registry–India, CTRI/2016/02/006678),^12^, ^13^ nested within this cross‐sectional study. This meant that the sample size was larger than required for the primary hypothesis outlined in our Introduction. For example, estimating that 20% of people would have completed high school, and ≈25% of people would have hypertension, provides adequate power (99%) to detect a difference between groups in those completing high school of as little as 4.1%.

### Ethics

This project was approved by each institutional ethics committee (Data S1) and the Health Ministry's Screening Committee of the Government of India (58/4/1F/CHR/2013/NCD II). Written informed consent was obtained from all participants before inclusion.

### Training

To ensure standardization, all field workers and supervisors were trained to measure anthropometric parameters and blood pressure (BP) and administer questionnaires, according to the World Health Organization STEPwise Approach to Surveillance protocol.^14^ Follow‐up training occurred ≈1 month after initial training to ensure consistency in data collection within and between sites.

### Clinical Measurements

Clinical measurements were made during the working day, mostly in the morning. Systolic BP (SBP) and diastolic BP were assessed using a digital automatic BP monitor (HEM–907; OMRON, Kyoto, Japan). Before measurement, participants sat quietly for 15 minutes, with legs uncrossed. BP was measured using the right upper arm, resting at the level of the heart, with a 3‐minute rest period between readings. At least 3 readings were recorded, with a fourth or fifth measurement taken when the final 2 measurements varied by ≥10 mm Hg SBP or ≥6 mm Hg diastolic BP. The mean of the last 2 measurements was used to determine BP.

Height was measured to the nearest 0.1 cm using a stadiometer (213; Seca, Hamburg, Germany), and weight to the nearest 0.1 kg using a digital weight scale (9000SV3R; Salter, Kent, UK). Waist circumference was measured horizontally at the midpoint between the iliac crest and the floating rib (after expiration), and hip circumference was measured at the fullest point of the buttocks, using a spring‐loaded tension tape (Gulick M‐22C; Patterson Medical, IL).

### Self‐Reported Data

Questionnaires were used to obtain information on lifestyle and SEP, the latter including education and household income. Annual household income in Indian rupees was obtained using a general question about estimated household income, supplemented by specific questions about income from rent of house, land, or equipment, as well as income from inheritance, investments, and gifts. The sum of these measures was then divided by the number of adults in the household to obtain an average individual income. Income was categorized into quartiles, with approximately similar number of participants in each group (Data S1). Participants also reported whether they held ration cards, and the type of ration card held.

Participants self‐reported their highest level of education completed and whether they could read and write. Educational attainment was categorized into 4 groups: no formal education, class 1 to 6, class 7 to 11, and completed class 12 or more.

Alcohol intake was recorded according to whether individuals had consumed any alcohol in the previous 30 days.

Hypertension was defined as having SBP ≥140 mm Hg, diastolic BP ≥90 mm Hg, and/or self‐reported use of BP‐lowering medication. Body mass index (BMI) was categorized as being overweight or obese (≥23 kg/m^2^) or normal (<23 kg/m^2^).^15^ Waist/hip ratio (WHR) was defined as being above normal when ≥0.8 for women and ≥0.9 for men. Regularity of physician visits and ease of access to health care were also documented (Data S1).

### Data Management

Hard copies of the questionnaires were scanned into tagged image files and distributed to the research group digitally. TeleForm Elite Version 9 software (Cardiff, San Jose, CA) was used to capture and verify the data in the tagged image files and export the data to a Microsoft Access database. All data were cleaned before analyses (Data S1).

### Statistical Analyses

All analyses were performed in Stata (Stata 15.0; College Station, TX). For continuous variables, we report means (SDs). Proportions were calculated for all categorical variables, and are presented as percentages. When variables were missing for a participant, that participant was excluded from any analyses involving that variable.

ANOVA was used to determine whether baseline characteristics of continuous variables differed between regions and by sex. Tukey's test was applied to determine which regions differed. Student unpaired *t* test was used to detect whether differences existed between women and men in each of the 3 regions. A Bonferroni correction was applied to protect against increased risk of type 1 error. Differences in categorical variables between regions and sex were analyzed using χ^2^ tests with Bonferroni correction to account for multiple comparisons between and within regions. Two‐tailed *P* values are reported.

Univariable and multivariable logistic regression analyses were used to measure the association between socioeconomic factors and hypertension. Multivariable analyses were initially adjusted for age, with age categorized into 3 groups, each group having approximately one third of the participants with hypertension. We then adjusted for income and education, both of which were dichotomized into the upper and lower 2 groups (as outlined above), to determine whether education modified the association between self‐reported income and hypertension. In these analyses, age was included as a continuous variable. To determine the interaction on an additive scale, we further assessed the relative excess risk caused by interaction, the attributable proportion, and the Synergy Index, using the technique described by VanderWeele and Knol.^16^ We also conducted sensitivity analyses of these associations, stratified by sex, age, and region. We further used logistic regression, adjusted for age and sex, to assess the association between SEP factors and having a WHR and BMI above normal, and consumption of alcohol in the past 30 days, and undertook similar analyses for relative excess risk caused by interaction, attributable proportion, and the Synergy Index as described above.

## Results

Among 16 949 people approached, 11 657 (68.8%) agreed to participate. The response rate was greatest in West Godavari (99%), least in Rishi Valley (45%), and intermediate in Trivandrum (77%). There were some differences in those who were recruited. For example, in the Rishi Valley region, the region with the poorest response rate, 30.5% of men aged 18 to 34.9 years agreed to participate, compared with 60.2% of men aged at least 65 years. In this same region, participation was also greater in women (50.3%) than in men (40.7%).

Participants in Trivandrum had a higher SEP, as indicated by the greater proportion of women and men who were able to read or write, and had completed at least class 12, than those in Godavari and Rishi Valley (Table 1). Overall, the proportion of men who could read and write was similar between Godavari and Rishi Valley, whereas women from Rishi Valley had the least educational attainment, with less than one third being able to read or write (Table 1).

**Table 1:** Age, BP, and Socioeconomic Characteristics of Participants in 3 Rural Regions in India, by Sex, 2014 to 2015

Characteristics	Rishi Valley		Godavari		Trivandrum		*P*_Region_	*P*_Sex_	*P*_Region×Sex_
	Women (n=1700)	Men (n=1700)	Women (n=2248)	Men (n=2231)	Women (n=1904)	Men (n=1853)			
Age, mean (SD), y	45.7 (16.5)	46.2 (16.7)	44.6 (17.1)	44.9 (17.5)	46.2 (17.4)	45.8 (18.0)	0.001^AC^	0.60	0.54
Age group, y							0.003^AB^	0.89	
18 to 34.9	500 (29.4)	500 (29.4)	752 (33.5)	739 (33.1)	604 (31.7)	615 (33.2)			
35 to 54.9	600 (35.3)	600 (35.3)	752 (33.5)	743 (33.3)	634 (33.3)	598 (32.3)			
≥55	600 (35.3)	600 (35.3)	744 (33.1)	749 (33.6)	666 (35.0)	640 (34.5)			
SBP, mean (SD), mm Hg	119.3 (20.0)^†^	124.7 (19.3)	119.2 (19.7)^†^	124.6 (17.3)	123.2 (20.1)^†^	127.3 (16.5)	<0.001^BC^	<0.001	0.18
DBP, mean (SD), mm Hg	72.7 (10.8)^†^	76.2 (11.9)	71.7 (11.6)^†^	75.3 (12.0)	72.8 (11.5)^†^	74.5 (11.5)	<0.001^AB^	<0.001	<0.001
SBP ≥140 mm Hg or DBP ≥90 mm Hg	263 (15.5)^†^ a	337 (19.9)^a^	351 (15.6)	402 (18.0)	383 (20.1)	375 (20.3)^a^	<0.001^BC^	0.002	
Hypertension	365 (21.5)^a^	414 (24.4)^a^	740 (32.9)*	637 (28.6)	705 (37.0)*	599 (32.3)	<0.001^D^	0.005	
Literacy									
Ability to read	534 (31.9)^†^ b	1102 (65.8)^b^	1317 (58.6)^†^	1432 (64.3)^a^	1656 (87.0)^†^	1777 (95.9)	<0.001^D^	<0.001	
Ability to write	502 (30.0)^†^ b	1040 (62.1)^b^	1182 (52.6)^†^	1358 (61.0)^a^	1620 (85.1)^†^	1755 (94.8)^a^	<0.001^D^	<0.001	
Highest level of schooling	^†^ d	c	^†^ a	a	^†^				
No formal education	853 (53.7)	309 (19.6)	703 (31.4)	501 (22.5)	291 (15.3)	107 (5.8)	<0.001^D^	<0.001	
Class 1 to 6	319 (20.1)	474 (30.1)	854 (38.2)	789 (35.5)	233 (12.2)	283 (15.3)			
Class 7 to 11	315 (19.8)	515 (32.7)	535 (23.9)	559 (25.2)	778 (40.9)	908 (49.0)			
Class ≥12	101 (6.4)	279 (17.7)	146 (6.5)	374 (16.8)	602 (31.6)	555 (30.0)			
Above poverty line or no ration card	113 (6.8)^b^	73 (4.4)^b^	233 (10.4)	209 (9.4)^a^	1191 (62.6)	1111 (60.0)^a^	<0.001^D^	0.007	
People in household	b	b							
Mean (SD)	4.4 (3.1)	4.6 (3.1)	3.7 (2.2)^†^	4.0 (2.1)	4.4 (1.9)	4.4 (1.9)^a^	<0.001^AC^	<0.001	0.07
≥5 People	692 (41.4)	679 (40.5)	620 (27.6)	636 (30.8)	811 (42.6)	761 (41.1)^a^	<0.001^AC^	0.61	
Income per adult per month	^†^ b	b	^†^ c	b	^†^ e	e			
Quartile 1, Rs 0 to 1000	1107 (66.2)	744 (44.4)	206 (9.8)	301 (13.6)	416 (36.9)	368 (29.2)	<0.001^D^	<0.001	
Quartile 2, Rs >1000 to 1900	199 (11.9)	362 (21.6)	447 (21.2)	591 (26.8)	252 (22.3)	275 (21.8)			
Quartile 3, Rs >1900 to 3000	184 (11.0)	262 (15.6)	803 (38.1)	839 (38.0)	199 (17.6)	274 (21.7)			
Quartile 4, Rs >3000	182 (10.9)	308 (18.4)	653 (31.0)	477 (21.6)	262 (23.2)	344 (27.3)			
Type of employment	*^b^	b	*^a^	a	*^a^	a			
Agricultural	754 (45.2)	894 (53.5)	411 (18.3)	1198 (53.7)	13 (0.7)	200 (10.8)	<0.001^D^	<0.001	
Nonagricultural	225 (13.5)	527 (31.6)	224 (10.0)	758 (34.0)	401 (21.1)	1220 (66.1)			
Unemployed	457 (27.4)	111 (6.7)	1612 (71.7)	262 (11.7)	1176 (61.9)	143 (7.7)			
Retired	234 (14.0)	138 (8.3)	···	13 (0.6)	311 (16.4)	284 (15.4)			
Visits to physician			^†^ a	a	^†^	a			
Never	1135 (67.8)	1119 (66.8)	427 (19.0)	834 (37.4)	253 (13.3)	875 (47.3)	<0.001^D^	<0.001	
Regular visits to physician	74 (4.4)	74 (4.4)	225 (10.0)	154 (6.9)	422 (22.2)	302 (16.3)			
Irregular, but visited within past year	285 (17.0)	283 (16.9)	1004 (44.7)	831 (37.3)	895 (47.0)	477 (25.8)			
Not visited in past 1 y	179 (10.7)	199 (11.9)	591 (26.3)	409 (18.4)	334 (17.5)	198 (10.7)			
Self‐reported difficulty in accessing health care	770 (46.0)*^b^	677 (40.4)^b^	796 (35.5)^†^ a	504 (22.6)^b^	201 (10.6)^†^	130 (7.0)	<0.001^D^	<0.001	

Hypertension refers to those with SBP ≥140 mm Hg, DBP ≥90 mm Hg, and/or self‐reported use of BP‐lowering medications. Data are presented as number (percentage) unless otherwise stated. *P*_Region_, *P*_Sex_, and *P*_Region×Sex_ were determined using ANOVA for continuous variables and χ^2^ test for categorical variables. For continuous variables, if *P*_Region_ ≤0.05, Tukey's test was used to determine which regions differed at *P*≤0.05. For categorical variables, χ^2^ test was used with a Bonferroni correction for multiple comparisons (3 regions). This is shown by superscript (A=Rishi Valley vs Godavari, B=Rishi Valley vs Trivandrum, C=Godavari vs Trivandrum, D=all differ). BP indicates blood pressure; DBP, diastolic BP; Rs, Indian rupee; SBP, systolic BP.

*P≤0.01, ^†^ *P*≤0.001 for differences in *P*_Sex_ or *P*_Region×Sex_ ≤0.05 between men and women, derived using Student unpaired *t* test or χ^2^ test, with Bonferroni correction for specific contrasts in each of the 3 regions. Class ≥12 includes individuals who graduated from secondary schooling, completed technical college, or completed university. Income level above the poverty line was assessed using self‐reported data for use of a government issued ration card.

a There are 1 to 15 missing observations.

b There are 16 to 34 missing observations.

c There are 123 to 139 missing variables.

d There are 212 missing observations.

e There are 592 to 775 missing observations.

The proportion of men with hypertension in Trivandrum was 3.7% greater than in Godavari and 7.9% greater than in Rishi Valley (Table 1). Women followed similar trends to men on mean SBP, and the proportion of people with hypertension (Table 1). A greater proportion of women had hypertension than men in both Godavari and Trivandrum, whereas a lesser proportion had hypertension in Rishi Valley (Table 1).

Residing in the higher SEP regions of Godavari or Trivandrum was associated with greater odds of hypertension than residing in the lowest SEP region, Rishi Valley (Table 2), with or without adjustment for age. This association remained when the analyses were stratified by 3 age groups (Table 2). Greater educational attainment appeared to be associated with lesser odds of hypertension (Table 2). However, people who were older tended to have lesser educational attainment than those who were younger, with age confounding the association between education and hypertension (Figure S1). When adjusted for the confounding effects of age, having some level of educational attainment was associated with greater odds of hypertension compared with having no formal education (Table 2), an association that remained when the analyses were stratified by age group. Compared with agricultural workers, nonagricultural workers were 53% more likely to have hypertension, whereas unemployed participants were 104% more likely to have hypertension, and retirees were 64% more likely to have hypertension. In analyses that were stratified by age group, it appeared that older unemployed people and all age categories of retirees were particularly vulnerable to having hypertension. Those in the highest quartile income bracket had 47% greater odds of hypertension than those in the lowest quartile (Table 2), although there was a large number of missing observations for income, particularly for those in Trivandrum (36.4%), and the characteristics of people with and without details on income were different for all variables (Table S1). The associations between these characteristics and hypertension were largely stronger for women than for men, although directionally similar (Tables S2 and S3).

**Table 2:** Factors Associated With Hypertension in 3 Rural Regions in India, 2014 to 2015: All Ages and Stratified by Age Group

Characteristics	Univariable			Adjusted for Age											
	All Ages (N=11 652)			All Ages (N=11 652)			18 to 34.9 y (N=3716)			35 to 54.9 y (N=3932)			≥55 y (N=4004)		
	OR	95% CI	*P* Value	OR	95% CI	*P* Value	OR	95% CI	*P* Value	OR	95% CI	*P* Value	OR	95% CI	*P* Value
Age, y	1.07	1.07 to 1.07	<0.001												
Age group, y															
18 to 34.9	1.00														
35 to 54.9	5.40	4.65 to 6.28	<0.001												
≥55	17.7	15.3 to 20.5	<0.001												
Women^a^	1.12	1.04 to 1.21	0.005	1.18	1.08 to 1.29	<0.001	0.58	0.44 to 0.76	<0.001	1.18	0.97 to 1.29	0.14	1.43	1.26 to 1.62	<0.001
Region															
Rishi Valley	1.00			1.00			1.00			1.00			1.00		
Godavari	1.49	1.35 to 1.65	<0.001	1.75	1.56 to 1.96	<0.001	2.08	1.40 to 3.10	<0.001	1.92	1.59 to 2.32	<0.001	1.58	1.35 to 1.84	<0.001
Trivandrum	1.79	1.61 to 1.98	<0.001	2.01	1.79 to 2.26	<0.001	2.69	1.81 to 4.00	<0.001	1.81	1.49 to 2.20	<0.001	2.11	1.80 to 2.48	<0.001
Literate: ability to write^b^	0.70	0.64 to 0.76	<0.001	1.34	1.22 to 1.47	<0.001	1.59	1.06 to 2.38	0.03	1.24	1.07 to 1.44	0.005	1.44	1.27 to 1.63	<0.001
Education^c^															
No formal education	1.00			1.00			1.00			1.00			1.00		
Class 1 to 6	0.87	0.78 to 0.97	0.01	1.20	1.07 to 1.36	0.003	1.16	0.56 to 2.42	0.69	1.25	1.02 to 1.53	0.03	1.17	1.00 to 1.36	0.05
Class 7 to 11	0.60	0.53 to 0.66	<0.001	1.48	1.30 to 1.67	<0.001	1.78	0.91 to 3.47	0.09	1.34	1.10 to 1.62	0.003	1.58	1.33 to 1.89	<0.001
Class ≥12	0.32	0.28 to 0.37	<0.001	1.43	1.21 to 1.69	<0.001	1.91	0.97 to 3.76	0.06	1.31	1.01 to 1.70	0.04	2.01	1.46 to 2.77	<0.001
Above poverty line or no ration card^b^	1.45	1.32 to 1.58	<0.001	1.48	1.33 to 1.63	<0.001	1.29	0.96 to 1.73	0.09	1.31	1.11 to 1.54	0.001	1.71	1.48 to 1.99	<0.001
At least 5 people living in household^b^	0.87	0.80 to 0.95	0.001	0.95	0.86 to 1.04	0.3	0.90	0.69 to 1.18	0.44	0.97	0.83 to 1.13	0.71	1.00	0.88 to 1.15	0.95
Type of employment^d^															
Agricultural	1.00			1.00			1.00			1.00			1.00		
Nonagricultural	0.91	0.81 to 1.02	0.12	1.53	1.35 to 1.74	<0.001	1.69	1.19 to 2.41	0.003	1.53	1.27 to 1.84	<0.001	1.51	1.22 to 1.87	<0.001
Unemployed	2.14	1.93 to 2.37	<0.001	2.04	1.82 to 2.28	<0.001	1.21	0.82 to 1.80	0.33	2.05	1.71 to 2.44	<0.001	2.66	2.26 to 3.15	<0.001
Retired	5.74	4.93 to 6.69	<0.001	1.64	1.39 to 1.94	<0.001	2.75	0.32 to 23.6	0.36	2.08	1.15 to 3.77	0.02	2.32	1.91 to 2.81	<0.001
Income per adult per month^e^															
Quartile 1, Rs 0 to 1000	1.00			1.00			1.00			1.00			1.00		
Quartile 2, Rs >1000 to 1900	0.96	0.85 to 1.09	0.6	1.22	1.06 to 1.39	0.005	1.71	1.11 to 2.64	0.02	1.30	1.04 to 1.61	0.02	1.01	0.84 to 1.22	0.93
Quartile 3, Rs >1900 to 3000	0.90	0.80 to 1.01	0.08	1.23	1.08 to 1.40	0.002	1.24	0.80 to 1.90	0.34	1.28	1.04 to 1.58	0.02	1.17	0.98 to 1.41	0.09
Quartile 4, Rs >3000	1.03	0.92 to 1.16	0.6	1.47	1.29 to 1.69	<0.001	1.76	1.16 to 2.65	0.007	1.29	1.04 to 1.61	0.02	1.62	1.33 to 1.98	<0.001
Visits to physician^b^															
Never	1.00			1.00			1.00			1.00			1.00		
Regular visits to physician	15.8	13.6 to 18.3	<0.001	8.05	6.86 to 9.44	<0.001	3.07	1.51 to 6.25	0.002	8.41	6.45 to 11.0	<0.001	8.10	6.52 to 10.1	<0.001
Irregular, but visited within past year	3.55	3.19 to 3.94	<0.001	2.61	2.33 to 2.92	<0.001	1.79	1.31 to 2.44	<0.001	2.61	2.17 to 3.16	<0.001	2.74	2.32 to 3.24	<0.001
Not visited in past 1 y	1.91	1.67 to 2.18	<0.001	1.55	1.34 to 1.79	<0.001	1.56	1.09 to 2.23	0.02	1.66	1.31 to 2.10	<0.001	1.44	1.17 to 1.77	0.001
Self‐reported difficulty in accessing health care^b^	1.01	0.93 to 1.11	0.79	0.87	0.79 to 0.97	0.01	0.76	0.55 to 1.07	0.11	1.03	0.87 to 1.21	0.74	0.81	0.71 to 0.93	0.003

Data are presented as odds ratio (95% CI). *P* values were generated using univariable and multivariable logistic regression. Hypertension is defined as a systolic blood pressure ≥140 mm Hg, a diastolic blood pressure ≥90 mm Hg, and/or taking blood pressure–lowering medication(s). Difficulty in accessing health care was defined as those reporting that access was fairly difficult or very difficult. Unemployed includes homemakers. Retired includes retirees and pensioners, regardless of whether they still report undertaking some agricultural activities. Nonagricultural indicates all forms of employment unrelated to agricultural work. N=11 652. OR indicates odds ratio; Rs, Indian rupee.

a There are 21 missing observations.

b There are 53 to 55 missing observations.

c There are 250 missing observations.

d There are 67 missing observations.

e There are 1579 missing observations.

The association between income and hypertension was similar when adjusted for age alone, or with an additional adjustment for education (Table 3). This pattern was similar for women and men (Tables S4 and S5), and was similar when using the lower cutoff for hypertension, as recommended by the 2017 American College of Cardiology/American Heart Association High Blood Pressure Guidelines (Table S6).^17^ Similarly, the association between level of education and hypertension did not appear to be modified appreciably by addition of income to the model, for either the whole sample (Table 3) or women and men separately (Tables S4 and S5), when using the cutoff of 130/80 mm Hg for defining hypertension (Table S6), or when stratified by region (Tables S6 and S7). With an additional adjustment for region, the odds ratios were reduced toward the null (Table S8).

**Table 3:** Association Between Income and/or Education and Hypertension and Its Risk Factors, 3 Rural Regions in India, 2014 to 2015

SEP Variable	Hypertension			WHR Above Normal^a^			BMI ≥23 kg/m^2^ b		
	OR	95% CI	*P* Value	OR	95% CI	*P* Value	OR	95% CI	*P* Value
Income per adult per month, adjusted for age									
Quartile 1, Rs 0 to 1000	1.00			1.00			1.00		
Quartile 2, Rs >1000 to 1900	1.21	1.06 to 1.39	0.006	1.73	1.53 to 1.94	<0.001	1.52	1.36 to 1.70	<0.001
Quartile 3, Rs >1900 to 3000	1.23	1.08 to 1.40	0.002	2.13	1.90 to 2.40	<0.001	1.90	1.71 to 2.12	<0.001
Quartile 4, Rs >3000	1.44	1.26 to 1.66	<0.001	2.41	2.13 to 2.73	<0.001	2.73	2.43 to 3.06	<0.001
Income per adult per month, adjusted for age and education									
Quartile 1, Rs 0 to 1000	1.00			1.00			1.00		
Quartile 2, Rs >1000 to 1900	1.19	1.03 to 1.37	0.02	1.66	1.47 to 1.88	<0.001	1.45	1.30 to 1.63	<0.001
Quartile 3, Rs >1900 to 3000	1.21	1.06 to 1.38	0.005	2.10	1.87 to 2.36	<0.001	1.87	1.68 to 2.09	<0.001
Quartile 4, Rs >3000	1.36	1.18 to 1.55	<0.001	2.22	1.96 to 2.51	<0.001	2.44	2.17 to 2.74	<0.001
Education, adjusted for age									
No formal education	1.00			1.00			1.00		
Class 1 to 6	1.20	1.06 to 1.37	0.005	1.79	1.58 to 2.02	<0.001	1.86	1.66 to 2.08	<0.001
Class 7 to 11	1.41	1.23 to 1.61	<0.001	2.23	1.97 to 2.52	<0.001	2.56	2.28 to 2.88	<0.001
Class ≥12	1.52	1.27 to 1.82	<0.001	2.22	1.91 to 2.57	<0.001	2.89	2.51 to 3.33	<0.001
Education, adjusted for age and income									
No formal education	1.00			1.00			1.00		
Class 1 to 6	1.16	1.02 to 1.32	0.025	1.63	1.44 to 1.84	<0.001	1.70	1.51 to 1.90	<0.001
Class 7 to 11	1.36	1.18 to 1.55	<0.001	2.11	1.86 to 2.39	<0.001	2.39	2.12 to 2.69	<0.001
Class ≥12	1.40	1.16 to 1.69	<0.001	1.98	1.70 to 2.31	<0.001	2.49	2.15 to 2.88	<0.001

n=9869 (1788 missing observations for education or income). Data are presented as odds ratio (95% CI). *P* values were generated using logistic regression, adjusted for age alone or adjusted for age and education/income. WHR above normal is defined as ≥0.8 for women and ≥0.9 for men. BMI indicates body mass index; OR, odds ratio; Rs, Indian rupee; SEP, socioeconomic position; WHR, waist/hip ratio.

a There are 62 additional missing observations.

b There are 24 additional missing observations.

In interaction analyses, there was no evidence that education modified the association between income and hypertension, or that income modified the association between education and hypertension, as shown by the interaction odds ratio, relative excess risk caused by interaction, attributable proportion, and Synergy Index (Table 4). This pattern was seen when stratified by age group (Table 4), in women and men separately (Table S9), when using the cut point of 130/80 mm Hg to define hypertension (Table S10), and when undertaking the analyses separately by region (Table S11).

**Table 4:** Association of Hypertension With Income and Education for Women and Men Combined, for All Age Groups and Stratified by Age, 3 Rural Regions in India, 2014 to 2015

Income per Adult per Month	Education Level								Measure of Effect Modification			
	No Education to Class 6				Class ≥7				On Additive Scale			
	N +/− Hypertension	OR	95% CI	*P* Value	N +/− Hypertension	OR	95% CI	*P* Value	Index	OR	95% CI	*P* Value
All ages												
Rs 0 to 1900	1016/1905	1.00			468/1785	1.38	1.19 to 1.60	<0.001				
Rs >1900	763/1535	1.28	1.12 to 1.45	<0.001	552/1845	1.53	1.33 to 1.77	<0.001				
									RERI	−0.12	−0.40 to 0.15	0.38
									AP	−0.08	−0.26 to 0.10	0.39
									SI	0.81	0.52 to 1.27	0.36
Age group 18 to 34.9 y												
Rs 0 to 1900	15/324	1.00			72/1103	1.72	0.97 to 3.05	0.07				
Rs >1900	25/444	1.21	0.63 to 2.34	0.56	90/1093	1.98	1.13 to 3.47	0.02				
									RERI	0.05	−0.86 to 0.97	0.91
									AP	0.02	−0.44 to 0.49	0.92
									SI	1.05	0.39 to 2.89	0.92
Age group 35 to 54.9 y												
Rs 0 to 1900	259/804	1.00			197/521	1.28	1.03 to 1.60	0.03				
Rs >1900	248/673	1.25	1.01 to 1.54	0.04	214/606	1.29	1.04 to 1.60	0.02				
									RERI	−0.24	−0.63 to 0.16	0.24
									AP	−0.18	−0.49 to 0.13	0.25
									SI	0.55	0.23 to 1.36	0.20
Age group ≥55 y												
Rs 0 to 1900	742/777	1.00			199/161	1.44	1.14 to 1.83	0.002				
Rs >1900	490/418	1.27	1.07 to 1.50	0.005	248/146	1.90	1.51 to 2.40	<0.001				
									RERI	0.19	−0.34 to 0.72	0.49
									AP	0.10	−0.17 to 0.37	0.47
									SI	1.26	0.64 to 2.49	0.50

Data are presented as odds ratio (95% CI), and all analyses are adjusted for age. n=9869 for all ages (1788 missing observations for education or income). n=3166 for those aged 18 to 34.9 years (551 missing observations for education or income). n=3522 for those aged 35 to 44.9 years (411 missing observations for education or income). n=3181 for those aged ≥55 years (824 missing observations for education or income). AP indicates attributable proportion; OR, odds ratio; Rs, Indian rupee; RERI, relative excess risk caused by interaction; SI, Synergy Index.

**Figure 2:**
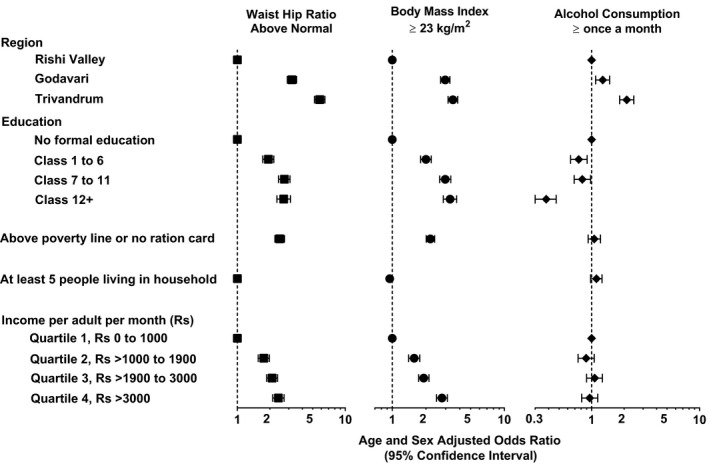
Socioeconomic factors associated with waist/hip ratio, body mass index, and alcohol consumption, 3 rural regions in India, 2014 to 2015. Data are presented as age‐ and sex‐adjusted odds ratios, and error bars indicate 95% CIs. Waist/hip ratio above normal is defined as ≥0.8 for women and ≥0.9 for men. Rs, Indian rupee.

Residing in Godavari (odds ratio, 3.20; 95% CI, 2.91–3.54) or Trivandrum (odds ratio, 5.80; 95% CI, 5.19–6.48; *P*<0.001) was associated with having a greater WHR than in Rishi Valley (Figure 2; Table S12). Similar trends were observed for BMI (Figure 2; Table S13). For each increasing category of educational attainment and income, there was an increased likelihood of having a BMI or WHR above normal values (all *P* for trend <0.001).

Income was positively associated with WHR, with each increasing category of income being associated with a greater likelihood of having a WHR above normal (*P* for trend <0.001; Table 3). A similar pattern was seen for education, although there may have been a threshold above class 6 education (Table 3), a pattern that was similar for BMI. However, there was no evidence for an interaction between education and income on WHR (Table S14). These findings appeared similar for women and men (Tables S4, S5, and S14) and were consistent across the 3 regions (Figure S2). Interestingly, there was evidence that education exacerbated the association between income and BMI ≥23 kg/m^2^ (Table S15).

Only 22 women (<0.4%) reported consuming alcohol in the previous month, and so the results for alcohol largely reflect patterns in men. Compared with Rishi Valley, living in Godavari was associated with 27% greater odds of consuming alcohol in the previous month, whereas living in Trivandrum was associated with an ≈2‐fold greater likelihood of consuming alcohol (Figure 2; Table S16; *P*<0.001). Completing any form of education was associated with reduced odds of consuming alcohol, whereas completing class 12+ was associated with 62% reduced likelihood of consuming alcohol in the previous month (*P*<0.001). There did not appear to be an association between indicators of income, such as possession of a below poverty line ration card or individual income per month, and alcohol consumption. Indeed, people in the highest categories of income appeared to consume alcohol at similar levels to those in the lowest quartiles (Figure 2; Table S16), although there did appear to be a difference by region (Figure S2). However, it does appear that, in men, education may modify the association between income and alcohol consumption, with a relative reduced risk caused by the interaction of 0.23, although this apparent effect was not statistically significant at conventional levels (Table S17).

People with hypertension were more likely to report taking medications for hypertension for each increasing category of educational attainment and income (*P*<0.001; Figure S3). This association was similar for women and men.

## Discussion

In 3 diverse rural sites across southern India, we found that higher SEP was associated with hypertension. Comparing between the sites, the prevalence of hypertension was greater in sites with higher average SEP. Overall, there was a positive association between measures of SEP and risk factors for hypertension, such as BMI and WHR, but not for alcohol consumption. These findings demonstrate that the positive association between SEP and hypertension may be fueled by adiposity in regions of higher SEP.

Education, and specifically health education, has been shown in some settings to mitigate the association between low SEP and hypertension,^18^, ^19^ potentially by modifying health behaviors.^19^ Our inability to detect a mitigating effect of education on the association between high income and hypertension in the setting of rural India may reflect a relative lack of health education within the curricula. Thus, targeted health education, in schools and in workplaces, may provide a pathway for controlling hypertension in regions that are rapidly undergoing urbanization and industrialization. Importantly, targeted health education in the community at large is also an important pathway that could be used to engage with the retirees and older unemployed people who appeared to be at particularly high risk of hypertension. Potentially, this strategy may be effective not only in India, but also in other settings where health education is suboptimal. Thus, randomized controlled trials of educational interventions, tailored to local cultural and socioeconomic conditions, are warranted. Notably, a recently completed cluster‐randomized controlled trial, conducted in the same 3 sites as the current study, demonstrated the effectiveness of a scalable group‐based education and monitoring program delivered by health workers for improving control of hypertension.^13^

The 3 regions of the study were at very different stages of the epidemiological transition, as shown by each measurement of SEP. Trivandrum was the most socioeconomically advanced, with almost all individuals able to read and write, fewer individuals in possession of a below poverty line ration card, and more individuals reporting income levels in the highest bracket. In contrast, in Rishi Valley, few did not possess a below poverty line ration card, and few had an income in the highest quartile, whereas the relative lack of education was largely limited to women. In all measures of SEP, Godavari was intermediate to Rishi Valley and Trivandrum. These data validate our use of region as a proxy measure of SEP.

When using region as a proxy for SEP, the proportion of people with hypertension was greater with each increasing level of SEP. Similar findings were evident when classifying SEP according to income, albeit in a slightly reduced sample size, and education. These findings are consistent with those of studies previously conducted in other sites of India and other low‐ to middle‐income countries, such as Uganda and China,^20^, ^21^, ^22^ but are in contradistinction to the findings from HICs, where greater educational attainment has been associated with a reduced risk of hypertension.^1^, ^23^

The difference in the association between SEP and hypertension in HICs versus low‐ to middle‐income countries may at least partly be attributable to the effects of epidemiological transition.^7^, ^24^ As transition progresses toward urbanization and industrialization, diets include a higher content of fat, and sedentary lifestyles are more common.^25^, ^26^ In these instances, socioeconomic factors play an important role in influencing the risk and outcome of noncommunicable conditions, such as hypertension, by affecting individuals’ ability to access and afford health care, lead healthy lifestyles, and take preventative measures.^24^

In HICs, at latter stages of the epidemiological transition, where sedentary lifestyles and access to high‐energy processed food persist, education may offer a mitigating effect on the risk of hypertension.^9^, ^27^ With increased educational attainment, there is increased knowledge about the risk factors for hypertension and measures to prevent high BP,^23^, ^26^ potentially influencing individuals to adopt healthier lifestyles.^7^, ^28^, ^29^ In the sample we studied, the relationships between education, risk factors for hypertension, and the risk of hypertension are not entirely clear. Although we found that higher SEP was associated with indicators of unhealthy lifestyle, such as greater BMI and greater WHR, higher educational attainment was associated with reduced odds of consuming alcohol in the past month. Therefore, it is likely that the positive relationship between SEP and hypertension may partly be fueled by some unhealthy lifestyle practices that are associated with SEP, although alcohol consumption does not appear to be among these. The fact that we did not find evidence for a mitigating effect of education on the association between income and hypertension or measures of adiposity, in our rural populations, leads us to speculate that people with higher SEP in rural India may lack health literacy.

Agricultural workers were less likely to be hypertensive than either nonagricultural workers or those who were unemployed or retired, even after adjustment for physical activity, sex, BMI, WHR, and age (data not shown). This indicates that there may be fundamental differences between agricultural workers and those who are nonagricultural workers or unemployed/retired, and that these differences may protect agricultural workers from the risk of hypertension. The precise nature of these differences remains to be determined.

Our finding that people with hypertension who had higher educational attainment were more likely to report taking antihypertensive medications may indicate better health literacy among this group than those with lower educational attainment. However, the fact that this trend for increasing use of medications was also observed with each greater level of income more likely points to greater access to health care and greater affordability of medications in those with more education and a higher income. As rural India is fast advancing along the epidemiological spectrum,^30^ the burden of hypertension in these populations is likely to increase substantially, so access to affordable medications will be critical to managing this increased burden.

A limitation of our study was the large proportion of people who refused to participate, particularly in one of the regions. Potentially, this may have biased the sample to those who were not working, as shown by the poorer response rates in men of working age than in those aged ≥65 years, or there may be other systematic biases that cannot be accounted for in the analysis. It is unclear whether this would have resulted in odds ratios that overestimated or underestimated the effect size. Importantly, the large proportion of people refusing to participate in the Rishi Valley region may have reduced the generalizability of our findings in this region. However, the fact that the findings are similar between the West Godavari region, the region with a 99% response rate, and the regions with poorer response rates somewhat mitigates this concern. A further limitation is the large proportion of missing data for participants’ income, with 13.6% of participants opting not to report their income, mostly in the highest SEP region. More important, we observed less educational attainment, lower levels of SBP, and lesser adiposity in those who chose to report their income than in those who refused. Thus, our data on income are not representative of the population sampled. This is likely to have biased the findings toward the null and may have reduced the likelihood of identifying potential modifications of education on the association of income and hypertension. Income was derived using self‐reported household income as well as details of additional rental and other income, and the sum of which was then divided by the number of adults in the household. Self‐reported income levels are potentially subject to serious measurement error, so the levels of income obtained may be inaccurate. The fact that we categorized income into quartiles somewhat reduces this potential bias. Furthermore, as income is a critical indicator of SEP, assessing the relationship between income, education, and hypertension in a more generalizable sample may provide clearer and more conclusive findings.

A major strength of our study is the large sample size of 11 657 participants from 3 diverse rural regions of India. This allowed collection of a relatively representative sample and enabled some generalizability to the population of interest. We also used rigorous training for all data collectors and research staff to ensure standardization of methods for data collection across the 3 sites. The questionnaires we administered were read aloud to participants to allow inclusion of participants irrespective of their ability to read or write. Together, these measures optimized the validity and generalizability of our findings.

In conclusion, the risk of hypertension was positively associated with higher SEP in rural India. In addition, modifiable risk factors, such as greater adiposity, were exacerbated with higher SEP. These modifiable risk factors may contribute to the increased risk of hypertension in people with higher SEP. In future studies, careful ascertainment of income, potentially by using a wealth index or determining what people spend and own rather than earn, and identifying where and how people learn about health, may provide further clarity about the relationship between SEP and hypertension in rural Indian populations, particularly if collected prospectively. In addition, comparing sites of higher SEP from urban regions with those from rural regions may also provide more information about the factors that influence the relationship between SEP and hypertension.

## Sources of Funding

This work was supported by the National Health and Medical Research Council (NHMRC; Australia; GNT 1040030) as part of the Global Alliance for Chronic Diseases Hypertension Program. Dr Thrift acknowledges fellowship support from the NHMRC (1042600). Dr Chow acknowledges an NHMRC Fellowship, cofunded by a Future Leader Fellowship (1105447) from the Heart Foundation (Australia). Dr Joshi acknowledges a Future Leader Fellowship (100484) from the Heart Foundation (Australia). Dr Guggilla is currently supported by a Research Fellowship from the Marie Sklodowska‐Curie Actions (European Union H2020 Grant 754432). The funders had no role in the design of the study, analyses, or decision to publish.

## Disclosures

Dr Chow reports grants from the National Health and Medical Research Council (NHMRC) and Heart Foundation; Drs Evans, Kalyanram, Kartik, and Thrift report grants from NHMRC for this study and for other projects outside the submitted work; Dr Guggilla reports research grants from the European Commission and the Polish Ministry of Science and Higher Education. Dr Guggilla holds shares in 3 Indian multinational pharmaceutical companies (Ajanta Pharma Limited, Divi's Laboratories Limited, and NATCO Pharma Limited). The remaining authors have no disclosures to report.

## Supporting information


**Data S1.** Supplemental methods.
**Table S1.** Baseline Characteristics According to Whether or Not Details of Income Were Provided, Three Rural Regions of India, 2014–2015
**Table S2.** Factors Associated With Hypertension in Women, Three Rural Regions in India, 2014–2015
**Table S3.** Factors Associated With Hypertension in Men, Three Rural Regions in India, 2014–2015
**Table S4.** Association Between Income and/or Education and Hypertension and Its Risk Factors in Women, Three Rural Regions in India, 2014–2015
**Table S5.** Association Between Income and/or Education and Hypertension and Its Risk Factors in Men, Three Rural Regions in India, 2014–2015
**Table S6.** Association Between Income and/or Education and Hypertension as Defined by a Cut‐Off of 130/80 mm Hg as Per the American College of Cardiology/American Heart Association Guidelines for Hypertension^5^; Three Rural Regions in India, 2014–2015
**Table S7.** Association Between Income and/or Education and Hypertension by Region, Three Rural Regions in India, 2014–2015
**Table S8.** Association Between Income and/or Education and Hypertension and its Risk Factors, Three Rural Regions in India, 2014–2015
**Table S9.** Modification of the Effect of Education on Hypertension by Income Level, Three Rural Regions in India, 2014–2015: by Sex
**Table S10.** Modification of the Effect of Education on Hypertension (130/80 mm Hg) by Income Level, Three Rural Regions in India, 2014–2015: Overall and by Sex
**Table S11.** Modification of the Effect of Education on Hypertension by Income Level for Women and Men Combined, 2014–2015: by Region
**Table S12.** Association Between SEP and Waist Hip Ratio Above Normal, Three Rural Regions in India, 2014–2015
**Table S13.** Association Between SEP and BMI ≥23 kg/m^2^, Three Rural Regions in India, 2014–2015
**Table S14.** Modification of the Effect of Education on Waist Hip Ratio Above Normal Levels, by Income Level, Three Rural Regions in India, 2014–2015: Overall and by Sex
**Table S15.** Women and Men Combined: Modification of the Effect of Education on BMI ≥23 kg/m^2^ by Income Level, Three Rural Regions in India, 2014–2015
**Table S16.** Association Between SEP and Alcohol Consumption in the Preceding 30 Days, Three Rural Regions in India, 2014–2015
**Table S17.** Men: Modification of the Effect of Education on Alcohol Use in the Past 30 Days by Income Level, Three Rural Regions in India, 2014–2015
**Figure S1.** Proportion of people in each category of age and education.
**Figure S2.** Association between Different Measures of SEP and Waist‐Hip Ratio Above Normal, Body Mass Index ≥23 kg/m^2^ and Alcohol Consumption at least once a month in Three Rural Regions in India, 2014–2015: (**A**) Rishi Valley; (**B**) Godavari; and (**C**) Trivandrum.
**Figure S3.** Proportion of people with hypertension reporting use of medications for hypertension in: (**A**) women and men (n=3310); (**B**) women (n=1687); and (**C**) men (n=1618).
