# Supplementary material for: Hypertension in Rural India: The Contribution of Socioeconomic Position
Source: J Am Heart Assoc. 2020 Mar 30;9(7):e014486. doi: 10.1161/JAHA.119.014486 (PMC7428634; doi:10.1161/JAHA.119.014486)
Supplement: Supplementary file 1 — Data S1. Supplemental methods. Table S1. Baseline Characteristics According to Whether or Not Details of Income Were Provided, Three Rural Regions of India, 2014–2015 Table S2. Factors Associated With Hypertension in Women, Three Rural Regions in India, 2014–2015 Table S3. Factors Associated With Hypertension in Men, Three Rural Regions in India, 2014–2015 Table S4. Association Between Income and/or Education and Hypertension and Its Risk Factors in Women, Three Rural Regions in India, 2014–2015 Table S5. Association Between Income and/or Education and Hypertension and Its Risk Factors in Men, Three Rural Regions in India, 2014–2015 Table S6. Association Between Income and/or Education and Hypertension as Defined by a Cut‐Off of 130/80 mm Hg as Per the American College of Cardiology/American Heart Association Guidelines for Hypertension5; Three Rural Regions in India, 2014–2015 Table S7. Association Between Income and/or Education and Hypertension by Region, Three Rural Regions in India, 2014–2015 Table S8. Association Between Income and/or Education and Hypertension and its Risk Factors, Three Rural Regions in India, 2014–2015 Table S9. Modification of the Effect of Education on Hypertension by Income Level, Three Rural Regions in India, 2014–2015: by Sex Table S10. Modification of the Effect of Education on Hypertension (130/80 mm Hg) by Income Level, Three Rural Regions in India, 2014–2015: Overall and by Sex Table S11. Modification of the Effect of Education on Hypertension by Income Level for Women and Men Combined, 2014–2015: by Region Table S12. Association Between SEP and Waist Hip Ratio Above Normal, Three Rural Regions in India, 2014–2015 Table S13. Association Between SEP and BMI ≥23 kg/m2, Three Rural Regions in India, 2014–2015 Table S14. Modification of the Effect of Education on Waist Hip Ratio Above Normal Levels, by Income Level, Three Rural Regions in India, 2014–2015: Overall and by Sex Table S15. Women and Men Combined: Modification of the Effect [file JAH3-9-e014486-s001.pdf]

SUPPLEMENTARY MATERIAL

Data S1. Supplementary Methods

Regions of the study

Trivandrum, located in Kerala is one of the most socioeconomically advantaged regions of India.[1] Seventy four percent of the population in Kerala reside in rural settings, yet changing patterns of employment and shifts towards the cultivation of cash crops have rendered Kerala the least agriculturally focused state in India.[1] Constituents of the District of Trivandrum often have access to healthcare and incomes in this region are high by Indian standards. The study site is within the Chirayinkeezhu Taluk, a rural region on the northern edge of the District of Trivandrum.

The West Godavari region (herein termed Godavari), located in northern Andhra Pradesh comprises 45 villages. Healthcare is less accessible in Godavari than Trivandrum. Average monthly household income is lower than in Trivandrum, with the majority of the residents of these regions working in agriculture or aquaculture.[2, 3]

The Rishi Valley region, located near the south-western border of Andhra Pradesh, encompasses approximately 240 hamlets. It is one of the poorest regions of India.[3] Most inhabitants of the Rishi Valley are subsistence farmers. Average household income is below internationally defined thresholds for poverty.[4]

Village leaders were contacted and informed about the proposed study, prior to commencement of the study.

Ethics

This project was approved by the Health Ministry's Screening Committee of the Government of India (58/4/1F/CHR/2013/NCD II), the Sree Chitra Tirunal Institute of Medical Sciences and Technology (SCT/IEC-484/July-2013), the Centre for Chronic Disease Control (CCDC-IEC-09-2012), Christian Medical College Vellore, and Monash University (CF13/2516 – 2013001327). Written informed consent was obtained from all participants prior to inclusion. When participants could not read or write, the patient information statement was read aloud to them, and consent recorded via a thumb print.

Questions on income, access to healthcare and employment

Income, in Indian Rupees, was recorded as household income. Household income was then divided by the number of adults in each household to generate income per person per month. Income was then categorized into four groups. The four categories were Rs 0 to 1000, Rs > 1000 to 1900, Rs > 1900 to 3000, Rs > 3000. The categories had approximately equal number of participants in the top

three quartiles. There were slightly more people in the bottom quartile because of the large number of people with an estimated income of Rs 1000.

Participants were asked how regularly they visited a doctor, with visits categorized as being regular, irregular but within the past 12 months, no visit within the previous year, or never. Access to healthcare was assessed by a general question about the ease of visiting a doctor according to five levels; difficulty comprised those reporting access to health care as being fairly difficult or very difficult.

Unemployed indicates those without employment, those seeking employment, beggars and homemakers. Retired includes retirees, ex-servicemen and pensioners. When individuals stated that they were a pensioner, but also undertook other activities, e.g. tending cattle, we deemed them as retired. Agricultural indicates farmers, farmhands, sericulture, aquaculture and farm machinery operators. People who indicated that they undertook both agricultural and nonagricultural activities were preferentially categorized as “agricultural” workers. Non-agricultural indicates all forms of employment unrelated to agricultural work including business owners, office workers, healthcare workers, manual laborers, students, and others.

Data cleaning

Data cleaning was performed in Stata (Stata 11.2, College Station, Texas, United States), with cleaned points corrected in two database locations as well as an audit trail. Data points were inspected for inconsistencies, such as extreme values, greatly variant blood pressure readings, or values inconsistent with adjacent parameters. Suspect data points were verified manually against the questionnaire and if necessary against on-site clinical records in India. Erroneous data points were replaced with correct values, or excluded where verified values were not available. Each parameter was inspected thoroughly before inclusion in analyses.

Table S1. Baseline Characteristics According to Whether or Not Details of Income Were Provided, Three Rural Regions of India, 2014–2015.

**Characteristics**	**Details provided on Income n = 10,075**	**No Details provided on Income n = 1,582**	***P***
Age (years), mean (SD)	45.3 (16.8)	47.1 (19.9)	<0.001
SBP (mmHg), mean (SD)	122.7 (18.8) *	125.0 (20.5) *	<0.001
DBP (mmHg), mean (SD)	74.1 (11.7) *	72.1 (11.6) *	<0.001
Hypertension	2,886 (28.7) *	580 (36.7) *	<0.001
Female	4,910 (48.8) †	942 (59.6) *	<0.001
Body Mass Index (kg/m^2), mean (SD)	23.1 (5.2) †	23.8 (5.6) *	<0.001
Waist Hip Ratio	0.89 (0.12) †	0.91 (0.08) †	<0.001
Site			
Rishi Valley	3,348 (33.2)	52 (3.3)	<0.001
Godavari	4,337 (43.1)	163 (10.3)	
Trivandrum	2,390 (23.7)	1,367 (86.4)	
Literacy rates			
Read	6,502 (64.6) *	1,328 (86.6) †	<0.001
Write	6,181 (61.4) *	1,286 (83.9) †	<0.001
Highest level of schooling	‡	†	
No formal education	2,536 (25.7)	233 (15.2)	<0.001
Class 1 to 6	2,684 (27.2)	275 (17.9)	
Class 7 to 11	3,048 (30.9)	568 (37.1)	
Class 12+	1,603 (16.2)	457 (29.8)	
Above poverty line or no ration card	2,039 (20.3) *	893 (58.3) †	<0.001
People in household			
Mean (SD)	4.2 (2.5)	4.3 (1.9) †	0.03
≥ 5 people	3,635 (36.1)	624 (40.8) †	<0.001
Visits to doctor	*	†	
Never	4,214 (41.9)	442 (28.8)	<0.001
Regular visits to doctor	960 (9.5)	292 (19.0)	
Irregular, but visited within past year	3,189 (31.7)	591 (38.5)	
Not visited in past 1 year	1,702 (16.9)	210 (13.7)	
Self-reported difficulty in accessing health care	2,913 (28.9) *	172 (11.2) †	<0.001

Abbreviations: DBP, diastolic blood pressure; Rs, Indian rupees; SBP, systolic blood pressure; SD, standard deviation.

Data are presented as number (%) unless otherwise stated. Income was missing for 36.4% of participants in Trivandrum, 3.6% in Godavari, and 1.5% in the Rishi Valley.

* 1-15 missing observations;

† 20-53 missing variables

‡ 104 missing variables

Table S2. Factors Associated with Hypertension in Women, Three Rural Regions in India, 2014–2015.

Characteristic	Univariable			Adjusted for Age		
	OR	95% CI	*P*	OR	95% CI	*P*
Age, years	1.08	1.08, 1.09	<0.001			
Age Group, years						
18-34.9	1.00					
35-54.9	7.50	5.94, 9.49	<0.001			
≥55	28.0	22.2, 35.2	<0.001			
Region						
Rishi Valley	1.00			1.00		
Godavari	1.79	1.55, 2.07	<0.001	2.32	1.96, 2.75	<0.001
Trivandrum	2.15	1.85, 2.49	<0.001	2.62	2.20, 3.12	<0.001
Literate: Ability to Write *	0.64	0.57, 0.71	<0.001	1.56	1.36, 1.79	<0.001
Education †						
No Formal Education	1.00			1.00		
Class 1 to 6	0.90	0.78, 1.04	0.16	1.61	1.36, 1.90	<0.001
Class 7 to 11	0.56	0.48, 0.64	<0.001	1.88	1.57, 2.25	<0.001
Class 12+	0.19	0.15, 0.24	<0.001	1.26	0.96, 1.67	0.1
Above poverty line or no ration card *	1.31	1.16, 1.48	<0.001	1.39	1.20, 1.61	<0.001
At least 5 people living in household *	0.95	0.84, 1.06	0.4	1.03	0.90, 1.18	0.7
Type of employment*						
Agricultural	1.00			1.00		
Non-agricultural	1.30	1.03, 1.65	0.03	1.85	1.42, 2.40	<0.001
Unemployed	3.15	2.63, 3.76	<0.001	2.81	2.32, 3.40	<0.001
Retired	10.1	7.95, 12.8	<0.001	2.06	1.58, 2.68	<0.001
Income per adult per month ‡						
Q1, Rs 0 to 1000	1.00			1.00		
Q2, Rs >1000 to 1900	1.03	0.86, 1.23	0.7	1.31	1.07, 1.61	0.009
Q3, Rs >1900 to 3000	1.00	0.85, 1.18	>0.9	1.54	1.27, 1.87	<0.001
Q4, Rs >3000	1.04	0.88, 1.23	0.7	1.61	1.32, 1.96	<0.001
Visits to doctor *						
Never	1.00			1.00		
Regular visits to doctor	22.1	17.7, 27.5	<0.001	13.2	10.4, 16.8	<0.001
Irregular, but visited within past year	4.25	3.60, 5.02	<0.001	3.62	3.01, 4.35	<0.001
Not visited in past 1 year	1.97	1.61, 2.41	<0.001	1.91	1.53, 2.40	<0.001
Self-reported difficulty in accessing health care *	1.04	0.92, 1.17	0.5	0.94	0.81, 1.08	0.4

Abbreviations: CI, confidence interval; OR, odds ratio; Q1-4, quartiles 1-4; Rs, rupees

N= 5,851. Data are presented as odds ratio (95% confidence interval). *P* values were generated using univariable and multivariable logistic regression. Hypertension is defined as a systolic blood pressure $\geq$ 140 mmHg and/or a diastolic blood pressure $\geq$ 90 mmHg and/or taking BP lowering medication(s). For difficulty in accessing healthcare, participants reported their level of difficulty according to five levels; difficulty comprised those reporting access to health care as being fairly difficult or very difficult. Unemployed indicates those without employment, those seeking employment, and homemakers. Retired refers to retirees, and pensioners. Agricultural indicates farmers, farmhands, and farm machinery operators. Non-agricultural indicates all forms of employment unrelated to agricultural work including business owners, office workers, healthcare workers, manual laborers, students, and others.

* 24-39 missing observations; † 121 missing observations; ‡ 941 missing observations.

Table S3. Factors Associated with Hypertension in Men, Three Rural Regions in India, 2014–2015.

Characteristic	Univariable			Adjusted for Age		
	**OR**	**95% CI**	***P***	**OR**	**95% CI**	***P***
Age, years	1.06	1.07, 1.07	<0.001			
Age Group, years						
18-34.9	1.00					
35-54.9	4.11	3.37, 5.00	<0.001			
≥55	11.9	9.80, 14.3	<0.001			
Region						
Rishi Valley	1.00			1.00		
Godavari	1.24	1.07, 1.43	0.004	1.36	1.16, 1.59	<0.001
Trivandrum	1.48	1.28, 1.72	<0.001	1.59	1.35, 1.87	<0.001
Literate: Ability to Write *	0.80	0.70, 0.90	<0.001	1.32	1.15, 1.51	<0.001
Education †						
No Formal Education	1.00			1.00		
Class 1 to 6	0.89	0.75, 1.06	0.2	1.06	0.88, 1.28	0.5
Class 7 to 11	0.68	0.57, 0.80	<0.001	1.40	1.16, 1.69	0.001
Class 12+	0.46	0.38, 0.56	<0.001	1.65	1.31, 2.08	<0.001
Above poverty line or no ration card *	1.60	1.41, 1.82	<0.001	1.57	1.36, 1.81	<0.001
At least 5 people living in household *	0.80	0.71, 0.90	0.001	0.87	0.76, 0.99	0.03
Type of employment *						
Agricultural	1.00			1.00		
Non-agricultural	0.78	0.68, 0.89	<0.001	1.31	1.13, 1.52	<0.001
Unemployed	3.16	2.60, 3.85	<0.001	1.71	1.37, 2.13	<0.001
Retired	4.27	3.45, 5.29	<0.001	1.50	1.19, 1.90	0.001
Income per adult per month ‡						
Q1, Rs 0 to 1000	1.00			1.00		
Q2, Rs >1000 to 1900	0.91	0.77, 1.07	0.3	1.14	0.95, 1.38	0.2
Q3, Rs >1900 to 3000	0.82	0.70, 0.97	0.02	1.04	0.87, 1.25	0.7
Q4, Rs >3000	1.02	0.86, 1.22	0.8	1.38	1.14, 1.67	0.001
Visits to doctor *						
Never	1.00			1.00		
Regular visits to doctor	11.8	9.53, 14.5	<0.001	5.51	4.41, 6.89	<0.001
Irregular, but visited within past year	3.20	2.78, 3.70	<0.001	2.16	1.85, 2.52	<0.001
Not visited in past 1 year	2.07	1.73, 2.49	<0.001	1.48	1.22, 1.80	<0.001
Self-reported difficulty in accessing health care *	0.95	0.83, 1.09	0.45	0.78	0.67, 0.91	0.001

Abbreviations: CI, confidence interval; OR, odds ratio; Q1-4, quartiles 1-4; Rs, rupees

N= 5,780. Data are presented as odds ratio (95% confidence interval). *P* values were generated using univariable and multivariable logistic regression. Hypertension is defined as a systolic blood pressure ≥ 140 mmHg and/or a diastolic blood pressure ≥ 90 mmHg and/or taking BP lowering medication(s). For difficulty in accessing healthcare, participants reported their level of difficulty according to five levels; difficulty comprised those reporting access to health care as being fairly difficult or very difficult. Unemployed indicates those without employment, those seeking employment, and homemakers. Retired refers to retirees, and pensioners. Agricultural indicates farmers, farmhands, and farm machinery operators. Non-agricultural indicates all forms of employment unrelated to agricultural work including business owners, office workers healthcare workers, manual laborers, students, and others.

* 23-46 missing observations; † 129 missing observations; ‡ 637 missing observations.

Table S4. Association Between Income and/or Education and Hypertension and its Risk Factors in Women, Three Rural Regions in India, 2014–2015.

SEP variable	Hypertension			WHR above Normal *			BMI ≥ 23 kg/m^2 †		
	OR	95% CI	*P*	OR	95% CI	*P*	OR	95% CI	*P*
**Income per adult per month, Adjusted for Age**									
Q1, Rs 0 to 1000	1.00			1.00			1.00		
Q2, Rs >1000 to 1900	1.31	1.06, 1.61	0.01	1.77	1.48, 2.10	<0.001	1.77	1.50, 2.09	<0.001
Q3, Rs >1900 to 3000	1.55	1.28, 1.88	<0.001	2.13	1.80, 2.50	<0.001	2.28	1.96, 2.66	<0.001
Q4, Rs >3000	1.59	1.30, 1.93	<0.001	2.39	2.02, 2.83	<0.001	2.88	2.46, 3.38	<0.001
**Income per adult per month, Adjusted for Age and Education**									
Q1, Rs 0 to 1000	1.00			1.00			1.00		
Q2, Rs >1000 to 1900	1.23	1.00, 1.52	0.05	1.66	1.39, 1.99	<0.001	1.63	1.38, 1.93	<0.001
Q3, Rs >1900 to 3000	1.48	1.22, 1.80	<0.001	2.12	1.79, 2.50	<0.001	2.22	1.90, 2.61	<0.001
Q4, Rs >3000	1.44	1.18, 1.77	<0.001	2.13	1.79, 2.53	<0.001	2.46	2.09, 2.91	<0.001
**Education, Adjusted for Age**									
No Formal Education	1.00			1.00			1.00		
Class 1 to 6	1.60	1.34, 1.92	<0.001	1.79	1.52, 2.11	<0.001	2.52	2.15, 2.95	<0.001
Class 7 to 11	1.78	1.46, 2.18	<0.001	2.55	2.14, 3.05	<0.001	3.62	3.06, 4.29	<0.001
Class 12+	1.26	0.92, 1.74	0.2	3.57	2.82, 4.50	<0.001	4.01	3.22, 5.00	<0.001
**Education, Adjusted for Age and Income**									
No Formal Education	1.00			1.00			1.00		
Class 1 to 6	1.49	1.23, 1.79	<0.001	1.58	1.33, 1.87	<0.001	2.19	1.86, 2.57	<0.001
Class 7 to 11	1.70	1.39, 2.09	<0.001	2.40	2.00, 2.87	<0.001	3.37	2.83, 4.00	<0.001
Class 12+	1.16	0.84, 1.61	0.4	3.30	2.60, 4.19	<0.001	3.60	2.87, 4.51	<0.001

Abbreviations: BMI, body mass index; CI, confidence interval; OR, odds ratio; Q1-4, quartiles 1-4; WHR, waist-hip ratio; Rs, rupees; SEP, socioeconomic position

n = 4,815 (1,037 missing observations for education or income); Data are presented as odds ratios (95% confidence interval). *P* values were generated using logistic regression adjusted for age alone, or adjusted for age and education/income.

* 42 additional missing observations; † 12 additional missing observations.

Table S5. Association Between Income and/or Education and Hypertension and its Risk Factors in Men, Three Rural Regions in India, 2014–2015.

SEP variable	Hypertension			WHR above Normal *			BMI ≥ 23 kg/m^2 *			Alcohol in Past 30 Days *		
	OR	95% CI	*P*	OR	95% CI	*P*	OR	95% CI	*P*	OR	95% CI	*P*
**Income per adult per month, Adjusted for Age**												
Q1, Rs 0 to 1000	1.00			1.00			1.00			1.00		
Q2, Rs >1000 to 1900	1.14	0.94, 1.38	0.2	1.76	1.49, 2.09	<0.001	1.41	1.20, 1.66	<0.001	0.90	0.75, 1.07	0.2
Q3, Rs >1900 to 3000	1.04	0.86, 1.25	0.7	2.18	1.84, 2.57	<0.001	1.69	1.45, 1.97	<0.001	1.06	0.90, 1.25	0.5
Q4, Rs >3000	1.35	1.12, 1.64	0.002	2.45	2.06, 2.93	<0.001	2.68	2.28, 3.16	<0.001	0.94	0.79, 1.12	0.5
**Income per adult per month, Adjusted for Age and Education**												
Q1, Rs 0 to 1000	1.00			1.00			1.00			1.00		
Q2, Rs >1000 to 1900	1.14	0.94, 1.38	0.2	1.78	1.50, 2.11	<0.001	1.42	1.21, 1.67	<0.001	0.90	0.75, 1.07	0.2
Q3, Rs >1900 to 3000	1.03	0.86, 1.24	0.7	2.21	1.87, 2.62	<0.001	1.72	1.47, 2.02	<0.001	1.06	0.90, 1.25	0.5
Q4, Rs >3000	1.24	1.01, 1.51	0.04	2.43	2.03, 2.91	<0.001	2.47	2.09, 2.92	<0.001	1.07	0.90, 1.29	0.4
**Education, Adjusted for Age**												
No Formal Education	1.00			1.00			1.00			1.00		
Class 1 to 6	1.05	0.86, 1.27	0.65	1.77	1.47, 2.13	<0.001	1.72	1.43, 2.05	<0.001	0.78	0.64, 0.93	0.007
Class 7 to 11	1.32	1.08, 1.61	0.006	2.03	1.68, 2.44	<0.001	2.47	2.06, 2.95	<0.001	0.81	0.67, 0.97	0.02
Class 12+	1.72	1.35, 2.19	<0.001	1.74	1.41, 2.16	<0.001	2.92	2.37, 3.59	<0.001	0.36	0.29, 0.45	<0.001
**Education, Adjusted for Age and Income**												
No Formal Education	1.00			1.00			1.00			1.00		
Class 1 to 6	1.04	0.86, 1.26	0.7	1.72	1.43, 2.08	<0.001	1.67	1.40, 2.00	<0.001	0.77	0.64, 0.93	0.006
Class 7 to 11	1.30	1.06, 1.58	0.01	2.01	1.66, 2.43	<0.001	2.37	1.98, 2.84	<0.001	0.80	0.67, 0.96	0.02
Class 12+	1.63	1.27, 2.09	<0.001	1.58	1.27, 1.97	<0.001	2.52	2.04, 3.12	<0.001	0.35	0.28, 0.45	<0.001

Abbreviations: BMI, body mass index; CI, confidence interval; OR, odds ratio; WHR, waist-hip ratio; Q1-4, quartiles 1-4; Rs, rupees; SEP, socioeconomic position

n = 5,034 (750 missing observations for education or income); Data are presented as odds ratios (95% confidence interval). *P* values were generated using logistic regression adjusted for age alone, or adjusted for age and education/income. * 12-15 additional missing observations

Table S6. Association Between Income and/or Education and Hypertension as defined by a cut-off of 130/80 mmHg as per the American College of Cardiology / American Heart Association guidelines for hypertension;[5] Three Rural Regions in India, 2014–2015.

SEP variable	Three Sites Combined			Rishi Valley			Godavari			Trivandrum		
	OR	95% CI	*P*	OR	95% CI	*P*	OR	95% CI	*P*	OR	95% CI	*P*
**Income per adult per month, Adjusted for Age**												
Q1, Rs 0 to 1000	1.00			1.00			1.00			1.00		
Q2, Rs >1000 to 1900	1.23	1.08, 1.39	0.001	1.18	0.95, 1.47	0.13	0.91	0.72, 1.15	0.44	1.17	0.92, 1.49	0.20
Q3, Rs >1900 to 3000	1.30	1.16, 1.46	<0.001	1.29	1.01, 1.65	0.04	1.00	0.80, 1.24	0.97	1.08	0.84, 1.39	0.54
Q4, Rs >3000	1.44	1.27, 1.63	<0.001	1.28	1.01, 1.61	0.04	1.09	0.86, 1.37	0.48	1.34	1.06, 1.69	0.02
**Income per adult per month, Adjusted for Age and Education**												
Q1, Rs 0 to 1000	1.00			1.00			1.00			1.00		
Q2, Rs >1000 to 1900	1.20	1.06, 1.35	0.004	1.16	0.93, 1.45	0.18	0.92	0.73, 1.17	0.50	1.15	0.90, 1.47	0.25
Q3, Rs >1900 to 3000	1.28	1.14, 1.44	<0.001	1.26	0.99, 1.62	0.06	1.01	0.81, 1.26	0.96	1.05	0.82, 1.36	0.69
Q4, Rs >3000	1.35	1.19, 1.52	<0.001	1.22	0.96, 1.56	0.10	1.07	0.84, 1.35	0.59	1.27	0.99, 1.62	0.06
**Education, Adjusted for Age**												
No Formal Education	1.00			1.00			1.00			1.00		
Class 1 to 6	1.27	1.13, 1.43	<0.001	1.16	0.95, 1.42	0.14	1.28	1.08, 1.51	0.004	0.80	0.54, 1.18	0.25
Class 7 to 11	1.42	1.26, 1.61	<0.001	1.19	0.96, 1.49	0.11	1.31	1.07, 1.59	0.009	1.08	0.77, 1.52	0.65
Class 12+	1.68	1.44, 1.95	<0.001	1.40	1.03, 1.90	0.03	1.50	1.16, 1.93	0.002	1.23	0.84, 1.79	0.29
**Education, Adjusted for Age and Income**												
No Formal Education	1.00			1.00			1.00			1.00		
Class 1 to 6	1.23	1.09, 1.38	0.001	1.13	0.93, 1.39	0.10	1.27	1.07, 1.50	0.005	0.78	0.53, 1.16	0.22
Class 7 to 11	1.38	1.22, 1.56	<0.001	1.15	0.92, 1.44	0.23	1.29	1.06, 1.58	0.01	1.04	0.74, 1.46	0.83
Class 12+	1.58	1.35, 1.85	<0.001	1.33	0.98, 1.81	0.07	1.47	1.14, 1.90	0.003	1.12	0.76, 1.66	0.57

Abbreviations: CI, confidence interval; OR, odds ratio; Q1-4, quartiles 1-4; Rs, rupees; SEP, socioeconomic position

n = 9,869 (1,788 missing observations for education or income); n = 3,160 Rishi Valley, n = 4,320 Godavari, n = 2,389 Trivandrum (missing observations for education or income: 236 Rishi Valley, 180 Godavari, 1,367 Trivandrum); Data are presented as odds ratios (95% confidence interval). *P* values were generated using logistic regression adjusted for age alone, or adjusted for age and education/income.

Table S7. Association Between Income and/or Education and Hypertension by Region, Three Rural Regions in India, 2014–2015.

SEP variable	Rishi Valley			Godavari			Trivandrum		
	OR	95% CI	*P*	OR	95% CI	*P*	OR	95% CI	*P*
**Income per adult per month, Adjusted for Age**									
Q1, Rs 0 to 1000	1.00			1.00			1.00		
Q2, Rs >1000 to 1900	1.00	0.77, 1.31	0.97	1.01	0.78, 1.30	0.97	1.20	0.92, 1.56	0.18
Q3, Rs >1900 to 3000	0.96	0.71, 1.31	0.80	1.06	0.83, 1.35	0.65	1.01	0.77, 1.34	0.93
Q4, Rs >3000	0.87	0.64, 1.19	0.38	1.28	0.99, 1.66	0.037	1.27	0.99, 1.64	0.06
**Income per adult per month, Adjusted for Age and Education**									
Q1, Rs 0 to 1000	1.00			1.00			1.00		
Q2, Rs >1000 to 1900	1.00	0.77, 1.31	0.99	1.01	0.78, 1.30	0.95	1.17	0.90, 1.52	0.24
Q3, Rs >1900 to 3000	0.95	0.70, 1.30	0.76	1.06	0.83, 1.35	0.65	0.99	0.74, 1.31	0.92
Q4, Rs >3000	0.84	0.61, 1.16	0.29	1.28	0.99, 1.66	0.055	1.22	0.93, 1.59	0.15
**Education, Adjusted for Age**									
No Formal Education	1.00			1.00			1.00		
Class 1 to 6	1.01	0.80, 1.27	0.95	1.23	1.03, 1.46	0.023	0.81	0.55, 1.18	0.27
Class 7 to 11	0.91	0.69, 1.20	0.53	1.32	1.06, 1.65	0.014	1.20	0.86, 1.67	0.29
Class 12+	1.30	0.88, 1.93	0.19	1.22	0.90, 1.66	0.19	1.21	0.83, 1.78	0.33
**Education, Adjusted for Age and Income**									
No Formal Education	1.00			1.00			1.00		
Class 1 to 6	1.02	0.80, 1.28	0.90	1.21	1.01, 1.44	0.037	0.80	0.54, 1.17	0.25
Class 7 to 11	0.93	0.70, 1.23	0.62	1.28	1.02, 1.60	0.032	1.16	0.83, 1.62	0.39
Class 12+	1.36	0.91, 2.03	0.13	1.16	0.85, 1.58	0.35	1.13	0.76, 1.69	0.55

Abbreviations: CI, confidence interval; OR, odds ratio; Q1-4, quartiles 1-4; Rs, rupees; SEP, socioeconomic position

n = 3,160 Rishi Valley, n = 4,320 Godavari, n = 2,389 Trivandrum (missing observations for education or income: 236 Rishi Valley, 180 Godavari, 1,367 Trivandrum); *P* values were generated using logistic regression adjusted for age alone, or adjusted for age and education/income.

Table S8. Association Between Income and/or Education and Hypertension and its Risk Factors, Three Rural Regions in India, 2014–2015.

SEP variable	Hypertension			WHR Above Normal *			BMI ≥ 23 kg/m^2 †		
	OR	95% CI	*P*	OR	95% CI	*P*	OR	95% CI	*P*
**Income per adult per month, Adjusted for Age and Region**									
Q1, Rs 0 to 1000	1.00	1.00		1.00			1.00		
Q2, Rs >1000 to 1900	1.09	0.95, 1.26	0.23	1.33	1.18, 1.52	<0.001	1.28	1.14, 1.44	<0.001
Q3, Rs >1900 to 3000	1.10	0.96, 1.26	0.18	1.59	1.40, 1.79	<0.001	1.58	1.42, 1.77	<0.001
Q4, Rs >3000	1.26	1.10, 1.45	0.001	1.81	1.59, 2.06	<0.001	2.26	2.01, 2.54	<0.001
**Income per adult per month, Adjusted for Age, Region and Education**									
Q1, Rs 0 to 1000	1.00	1.00		1.00			1.00		
Q2, Rs >1000 to 1900	1.09	0.94, 1.25	0.25	1.31	1.16, 1.49	<0.001	1.27	1.13, 1.43	<0.001
Q3, Rs >1900 to 3000	1.10	0.96, 1.26	0.19	1.56	1.38, 1.76	<0.001	1.60	1.43, 1.79	<0.001
Q4, Rs >3000	1.25	1.08, 1.44	0.002	1.77	1.55, 2.02	<0.001	2.16	1.92, 2.43	<0.001
**Education, Adjusted for Age and Region**									
No Formal Education	1.00	1.00		1.00			1.00		
Class 1 to 6	1.11	0.97, 1.26	0.12	1.50	1.32, 1.69	<0.001	1.65	1.47, 1.86	<0.001
Class 7 to 11	1.12	0.97, 1.30	0.11	1.44	1.26, 1.64	<0.001	1.89	1.67, 2.13	<0.001
Class 12+	1.16	0.96, 1.41	0.12	1.19	1.01, 1.39	0.04	1.94	1.67, 2.25	<0.001
**Education, Adjusted for Age, Region and Income**									
No Formal Education	1.00	1.00		1.00			1.00		
Class 1 to 6	1.09	0.95, 1.24	0.22	1.42	1.25, 1.61	<0.001	1.56	1.39, 1.75	<0.001
Class 7 to 11	1.10	0.96, 1.28	0.18	1.41	1.23, 1.61	<0.001	1.83	1.62, 2.07	<0.001
Class 12+	1.11	0.91, 1.34	0.30	1.14	0.96, 1.34	0.13	1.77	1.52, 2.07	<0.001

Abbreviations: BMI, body mass index; CI, confidence interval; OR, odds ratio; Q1-Q4, quartiles 1-4; Rs, Indian rupees; WHR, waist-hip ratio.
n = 9,869 (1,788 missing observations for education or income); Data are presented as odds ratios (95% confidence interval). *P* values were generated using logistic regression adjusted for age and region, or adjusted for age, region and education/income. WHR above normal is defined as ≥0.8 for women and ≥0.9 for men.
* 62 additional missing observations; † 24 additional missing observations

Table S9. Modification of the Effect of Education on Hypertension by Income Level, Three Rural Regions in India, 2014–2015: by Sex.

Income per adult per month	Education Level								Measure of Effect Modification on Additive Scale			
	No Education to Class 6				Class 7 and Above							
	N +/- HTN	OR	95% CI	*P*	N +/- HTN	OR	95% CI	*P*	Index	OR	95% CI	*P*
**Women**												
Rs 0 to 1900	570 / 1,062	1.00			174 / 772	1.49	1.18, 1.87	<0.001				
Rs >1900	451 / 817	1.53	1.28, 1.83	< 0.001	193 / 776	1.78	1.42, 2.24	<0.001				
									RERI	-0.23	-0.72, 0.25	0.35
									AP	-0.13	-0.42, 0.15	0.37
									SI	0.77	0.45, 1.31	0.34
**Men**												
Rs 0 to 1900	442 / 838	1.00			294 / 1,011	1.37	1.12, 1.67	0.002				
Rs >1900	312 / 716	1.07	0.88, 1.29	0.51	358 / 1,063	1.48	1.22, 1.79	<0.001				
									RERI	0.05	-0.29, 0.38	0.79
									AP	0.03	-0.20, 0.26	0.79
									SI	1.11	0.51, 2.40	0.80

Abbreviations: AP, Attributable proportion; CI, confidence interval; HTN, hypertension; OR, odds ratio; RERI, Relative Excess Risk due to Interaction; Rs, rupees; SI, Synergy Index

Data are presented as odds ratios (95% confidence interval), and all analyses are adjusted for age.

n = 4,815 for women (1,037 missing observations for education or income)

n = 5,034 for men (750 missing observations for education or income)

Table S10. Modification of the Effect of Education on Hypertension (130/80 mmHg) by Income Level, Three Rural Regions in India, 2014–2015: Overall and by Sex.

Income per adult per month	Education Level								Measure of Effect Modification on Additive Scale			
	No Education to Class 6				Class 7 and Above							
	N +/- HTN	OR	95% CI	*P*	N +/- HTN	OR	95% CI	*P*	Index	OR	95% CI	*P*
**Women and Men Combined**												
Rs 0 to 1900	1,443 / 1,478	1.00			803 / 1,450	1.37	1.20, 1.56	<0.001				
Rs >1900	1,129 / 1,169	1.31	1.16, 1.47	<0.001	953 / 1,444	1.59	1.40, 1.81	<0.001				
									RERI	-0.08	-0.32, 0.16	0.52
									AP	-0.05	-0.20, 0.10	0.53
									SI	0.88	0.61, 1.27	0.51
**Women**												
Rs 0 to 1900	775 / 857	1.00			277 / 669	1.47	1.20, 1.81	<0.001				
Rs >1900	614 / 654	1.52	1.29, 1.80	<0.001	286 / 683	1.54	1.26, 1.89	<0.001				
									RERI	-0.45	-0.87, -0.04	0.03
									AP	-0.29	-0.58, -0.01	0.05
									SI	0.55	0.32, 0.93	0.03
**Men**												
Rs 0 to 1900	663 / 617	1.00			526 / 779	1.23	1.04, 1.47	0.02				
Rs >1900	513 / 515	1.13	0.95, 1.35	0.16	664 / 757	1.54	1.30, 1.82	<0.001				
									RERI	0.17	-0.11, 0.46	0.23
									AP	0.11	-0.07, 0.29	0.23
									SI	1.47	0.68, 3.18	0.33

Abbreviations: AP, Attributable proportion; CI, confidence interval; HTN, hypertension; OR, odds ratio; RERI, Relative Excess Risk due to Interaction; Rs, Rupees; SI, Synergy Index
Data are presented as odds ratios (95% confidence interval), and all analyses are adjusted for age.
n = 9,869 for women and men combined (1,788 missing observations for education or income)
n = 4,815 for women (1,037 missing observations for education or income)
n = 5,034 for men (750 missing observations for education or income)

Table S11. Modification of the Effect of Education on Hypertension by Income Level for Women and Men Combined, 2014–2015: by Region.

Income per adult per month	Education Level								Measure of Effect Modification on Additive Scale			
	No Education to Class 6				Class 7 and Above							
	N +/- HTN	OR	95% CI	*P*	N +/- HTN	OR	95% CI	*P*	Index	OR	95% CI	*P*
**Rishi Valley**												
Rs 0 to 1900	451 / 1,080	1.00			92 / 695	0.96	0.72, 1.27	0.75				
Rs >1900	81 / 339	0.85	0.64, 1.14	0.29	55 / 367	0.98	0.70, 1.38	0.91				
									RERI	0.17	-0.28, 0.62	0.46
									AP	0.17	-0.26, 0.60	0.43
									SI	–	–	–
**Godavari**												
Rs 0 to 1900	384 / 633	1.00			97 / 431	1.34	0.99, 1.80	0.06				
Rs >1900	611 / 1,124	1.22	1.02, 1.46	0.03	216 / 824	1.29	1.02, 1.63	0.03				
									RERI	-0.27	-0.72, 0.19	0.25
									AP	-0.21	-0.56, 0.15	0.26
									SI	0.52	0.20, 1.39	0.19
**Trivandrum**												
Rs 0 to 1900	181 / 192	1.00			279 / 659	1.29	0.97, 1.71	0.08				
Rs >1900	71 / 72	0.91	0.59, 1.39	0.65	281 / 654	1.37	1.03, 1.83	0.03				
									RERI	0.18	-0.30, 0.66	0.46
									AP	0.13	-0.22, 0.48	0.47
									SI	1.93	0.13, 28.4	0.63

Abbreviations: AP, Attributable proportion; CI, confidence interval; HTN, hypertension; OR, odds ratio; RERI, Relative Excess Risk due to Interaction; Rs, Rupees; SI, Synergy Index

Data are presented as odds ratios (95% confidence interval), and all analyses are adjusted for age.

n = 3,160 for the Rishi Valley (236 missing observations for education or income)

n = 4,320 for Godavari (180 missing observations for education or income)

n = 2,389 for Trivandrum (1,367 missing observations for education or income)

Table S12. Association between SEP and Waist Hip Ratio Above Normal, Three Rural Regions in India, 2014–2015.

Characteristic	Univariable			Adjusted for Age and Sex		
	**OR**	**95% CI**	***P***	**OR**	**95% CI**	***P***
Region						
Rishi Valley	1.00			1.00		
Godavari	2.83	2.58, 3.11	<0.001	3.20	2.91, 3.54	<0.001
Trivandrum	5.13	4.62, 5.71	<0.001	5.80	5.19, 6.48	<0.001
Education *						
No Formal Education	1.00			1.00		
Class 1 to 6	1.37	1.23, 1.53	<0.001	1.94	1.72, 2.18	<0.001
Class 7 to 11	1.32	1.19, 1.46	<0.001	2.73	2.41, 3.08	<0.001
Class 12+	0.90	0.80, 1.02	0.09	2.69	2.33, 3.11	<0.001
Above poverty line or no ration card †	2.42	2.19, 2.68	<0.001	2.47	2.23, 2.74	<0.001
At least 5 people living in household †	0.91	0.84, 0.99	0.03	0.97	0.89, 1.05	0.40
Income per adult per month ‡						
Q1, Rs 0 to 1000	1.00			1.00		
Q2, Rs >1000 to 1900	1.53	1.36, 1.71	<0.001	1.76	1.56, 1.98	<0.001
Q3, Rs >1900 to 3000	1.76	1.58, 1.96	<0.001	2.10	1.87, 2.36	<0.001
Q4, Rs >3000	2.00	1.78, 2.25	<0.001	2.40	2.13, 2.71	<0.001

Abbreviations: CI, confidence interval; OR, odds ratio; Q1-4, quartiles 1-4; Rs, rupees; SEP, socioeconomic position

n =11,576 for univariable and n=11,563 for adjusted analyses. Waist hip ratio above normal is defined as ≥0.8 for women and ≥0.9 for men. *P* values were generated using univariable and multivariable logistic regression. Class 12+ includes individuals who graduated from secondary schooling, completed technical college or completed university. Income level above the poverty line was assessed using self-reported data for use of a government issued ration card.

* 238 missing observations. † 37-43 missing observations. ‡ 1,564-1,565 missing observations.

Table S13. Association between SEP and BMI ≥23 kg/m^2, Three Rural Regions in India, 2014–2015.

Characteristic	Univariable			Adjusted for Age and Sex		
	**OR**	**95% CI**	***P***	**OR**	**95% CI**	***P***
Region						
Rishi Valley	1.00			1.00		
Godavari	2.91	2.65, 3.20	<0.001	2.97	2.70, 3.27	<0.001
Trivandrum	3.45	3.13, 3.81	<0.001	3.48	3.15, 3.84	<0.001
Education *						
No Formal Education	1.00			1.00		
Class 1 to 6	1.62	1.46, 1.80	<0.001	2.00	1.79, 2.23	<0.001
Class 7 to 11	1.99	1.80, 2.21	<0.001	2.97	2.65, 3.33	<0.001
Class 12+	1.84	1.64, 2.06	<0.001	3.28	2.86, 3.76	<0.001
Above poverty line or no ration card †	2.21	2.03, 2.41	<0.001	2.19	2.01, 2.39	<0.001
At least 5 people living in household †	0.94	0.87, 1.01	0.09	0.95	0.88, 1.02	0.20
Income per adult per month ‡						
Q1, Rs 0 to 1000	1.00			1.00		
Q2, Rs >1000 to 1900	1.48	1.32, 1.66	<0.001	1.57	1.40, 1.76	<0.001
Q3, Rs >1900 to 3000	1.81	1.62, 2.01	<0.001	1.91	1.72, 2.13	<0.001
Q4, Rs >3000	2.63	2.35, 2.94	<0.001	2.78	2.48, 3.11	<0.001

Abbreviations: BMI, body mass index; CI, confidence interval; OR, odds ratio; Q1-4, quartiles 1-4; Rs, rupees; SEP, socioeconomic position

n =11,616 for univariable and n=11,595 for adjusted analyses. *P* values were generated using univariable and multivariable logistic regression. Class 12+ includes individuals who graduated from secondary schooling, completed technical college or completed university. Income level above the poverty line was assessed using self-reported data for use of a government issued ration card.

* 240 missing observations. † 41-45 missing observations. ‡ 1,567 – 1,568 missing observations.

Table S14. Modification of the Effect of Education on Waist Hip Ratio Above Normal Levels, by Income Level, Three Rural Regions in India, 2014–2015: Overall and by Sex.

Income per adult per month	Education Level: No Education to Class 6				Education Level: Class 7 and Above				Measure of Effect Modification on Additive Scale			
	**N +/- WHR above normal**	**OR**	**95% CI**	***P***	**N +/- WHR above normal**	**OR**	**95% CI**	***P***	**Index**	**OR**	**95% CI**	***P***
**Women and Men Combined**												
Rs 0 to 1900	1,734 / 1,160	1.00			1,314 / 920	1.82	1.60, 2.07	<0.001				
Rs >1900	1,631 / 662	2.07	1.83, 2.34	<0.001	1,648 / 738	2.82	2.48, 3.21	<0.001				
									RERI	-0.07	-0.43, 0.30	0.72
									AP	-0.02	-0.15, 0.11	0.72
									SI	0.96	0.79, 1.17	0.72
**Women**												
Rs 0 to 1900	925 / 686	1.00			600 / 334	2.53	2.09, 3.06	<0.001				
Rs >1900	893 / 372	2.20	1.87, 2.60	<0.001	694 / 269	3.69	3.03, 4.49	<0.001				
									RERI	-0.04	-0.74, 0.66	0.90
									AP	-0.01	-0.20, 0.18	0.90
									SI	0.98	0.76, 1.27	0.90
**Men**												
Rs 0 to 1900	805 / 472	1.00			714 / 586	1.41	1.18, 1.69	<0.001				
Rs >1900	737 / 290	1.90	1.58, 2.29	<0.001	950 / 468	2.30	1.92, 2.75	<0.001				
									RERI	-0.01	-0.44, 0.41	0.95
									AP	-0.01	-0.19, 0.18	0.95
									SI	0.99	0.71, 1.37	0.95

Abbreviations: AP, Attributable proportion; CI, confidence interval; OR, odds ratio; RERI, Relative Excess Risk due to Interaction; Rs, Rupees; SI, Synergy Index; WHR, waist to hip ratio
Data are presented as odds ratios (95% confidence interval), and all analyses are adjusted for age.
n = 9,805 for women and men combined (1,850 missing observations for education, income or WHR)
n = 4,773 for women (1,079 missing observations for education, income, or WHR)
n = 5,022 for men (762 missing observations for education, income, or WHR)

Table S15. Women and Men Combined: Modification of the Effect of Education on BMI ≥ 23 kg/m^2 by Income Level, Three Rural Regions in India, 2014–2015.

Income per adult per month	Education Level								Measure of Effect Modification on Additive Scale			
	No Education to Class 6				Class 7 and Above							
	**N +/- BMI ≥ 23 kg/m^2**	**OR**	**95% CI**	***P***	**N +/- BMI ≥ 23 kg/m^2**	**OR**	**95% CI**	***P***	**Index**	**OR**	**95% CI**	***P***
**Women and Men Combined**												
Rs 0 to 1900	1,045 / 1,863	1.00			1,030 / 1,215	1.94	1.72, 2.19	<0.001				
Rs >1900	1,159 / 1,137	1.96	1.75, 2.20	<0.001	1,440 / 956	3.42	3.03, 3.86	<0.001				
									RERI	0.52	0.15, 0.88	0.006
									AP	0.15	0.05, 0.25	0.003
									SI	1.27	1.07, 1.51	0.007
**Women**												
Rs 0 to 1900	610 / 1,013	1.00			488 / 457	2.69	2.24, 3.23	<0.001				
Rs >1900	707 / 559	2.39	2.05, 2.79	<0.001	629 / 340	4.72	3.91, 5.68	<0.001				
									RERI	0.63	-0.14, 1.41	0.11
									AP	0.13	-0.02, 0.28	0.08
									SI	1.21	0.96, 1.51	0.10
**Men**												
Rs 0 to 1900	432 / 844	1.00			541 / 757	1.66	1.40, 1.97	<0.001				
Rs >1900	451 / 577	1.61	1.36, 1.91	<0.001	809 / 611	3.04	2.57, 3.59	<0.001				
									RERI	0.77	0.35, 1.19	<0.001
									AP	0.25	0.13, 0.38	<0.001
									SI	1.60	1.20, 2.15	0.002

Abbreviations: AP, Attributable proportion; BMI, body mass index; CI, confidence interval; OR, odds ratio; RERI, Relative Excess Risk due to Interaction; Rs, Rupees; SI, Synergy Index
Data are presented as odds ratios (95% confidence interval), and all analyses are adjusted for age.
n = 9,845for women and men combined (1,812 missing observations for education, income or WHR)
n = 4,803 for women (1,049 missing observations for education, income, or BMI)
n = 5,022 for men (762 missing observations for education, income, or BMI)

Table S16. Association between SEP and Alcohol Consumption in the Preceding 30 days, Three Rural Regions in India, 2014–2015.

Characteristic	Univariable			Adjusted for Age and Sex		
	**OR**	**95% CI**	***P***	**OR**	**95% CI**	***P***
Region						
Rishi Valley	1.00			1.00		
Godavari	1.24	1.08, 1.42	0.003	1.27	1.09, 1.47	0.002
Trivandrum	1.84	1.61, 2.11	<0.001	2.12	1.82, 2.46	<0.001
Education *						
No Formal Education	1.00			1.00		
Class 1 to 6	1.40	1.20, 1.64	<0.001	0.76	0.64, 0.91	0.003
Class 7 to 11	1.71	1.48, 1.98	<0.001	0.82	0.69, 0.98	0.03
Class 12+	1.02	0.85, 1.22	0.80	0.38	0.30, 0.47	<0.001
Above poverty line or no ration card †	0.97	0.86, 1.10	0.70	1.06	0.93, 1.21	0.40
At least 5 people living in household†	1.11	1.00, 1.24	0.05	1.11	0.98, 1.25	0.09
Income per adult per month ‡						
Q1, Rs 0 to 1000	1.00			1.00		
Q2, Rs >1000 to 1900	1.23	1.05, 1.44	0.01	0.89	0.75, 1.06	0.20
Q3, Rs >1900 to 3000	1.32	1.14, 1.53	<0.001	1.07	0.90, 1.26	0.40
Q4, Rs >3000	1.13	0.97, 1.33	0.10	0.96	0.81, 1.14	0.70

Abbreviations: CI, confidence interval; OR, odds ratio; Q1-4, quartiles 1-4; Rs, rupees; SEP, socioeconomic position

n =11,568 for univariable and n=11,547 for adjusted analyses. *P* values were generated using univariable and multivariable logistic regression. Class 12+ includes individuals who graduated from secondary schooling, completed technical college or completed university. Income level above the poverty line was assessed using self-reported data for use of a government issued ration card.

* 201 missing observations. † 1-5 missing observations. ‡ 1,526-1,527 missing observations.

Table S17. Men: Modification of the Effect of Education on Alcohol Use in the Past 30 Days by Income Level, Three Rural Regions in India, 2014–2015.

Income per adult per month	Education Level: No Education to Class 6 N = 2,301				Class 7 and Above N = 2,718			
	**N +/- Alcohol in past 30 days**	**OR**	**95% CI**	***P***	**N +/- Alcohol in past 30 days**	**OR**	**95% CI**	***P***
Rs 0 to 1900	349 / 927	1.00			349 / 951	0.84	0.70, 1.02	0.07
Rs >1900	328 / 697	1.20	1.00, 1.44	0.05	367 / 1,051	0.81	0.68, 0.98	0.03

Abbreviations: CI, confidence interval; OR, odds ratio; Rs, rupees

n = 5,019 (765 missing observations for education, income, or alcohol consumption); Data are presented as odds ratios (95% confidence interval), and all analyses are adjusted for age.

Measure of effect modification on additive scale:

Relative Excess Risk due to Interaction (RERI) and (95% CI) = -0.23 (-0.49, 0.03), *P* = 0.09

Attributable proportion (AP) and (95% CI) = -0.28 (-0.60, 0.03), *P* = 0.08

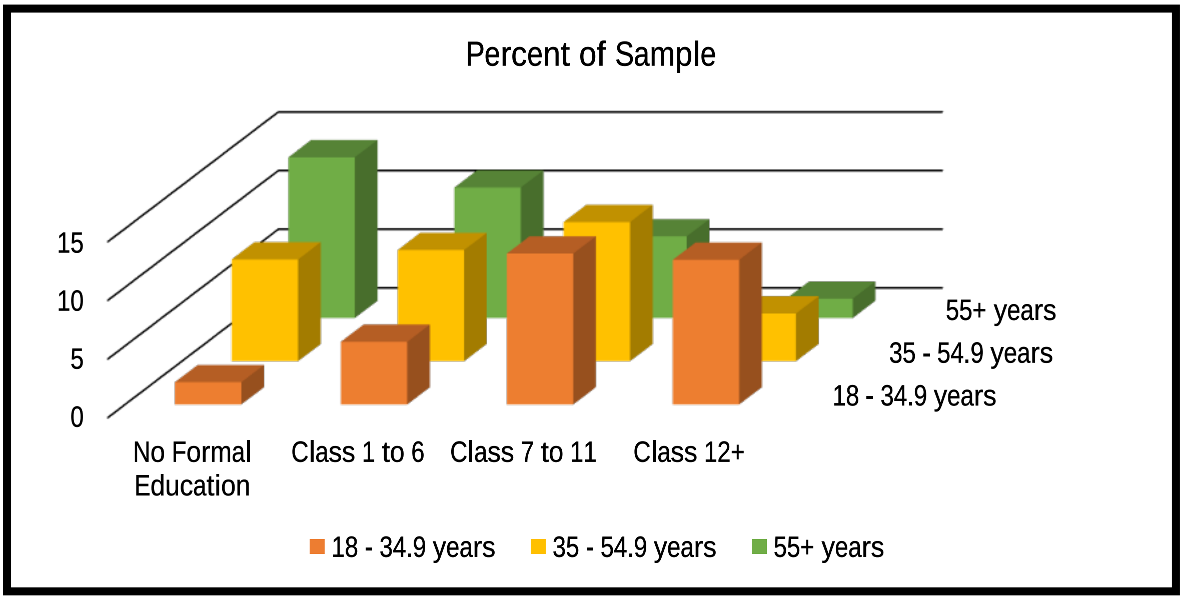

Figure S1. Proportion of people in each category of age and education.

Class 12+ includes individuals who graduated from secondary schooling, completed technical college or completed university.

Age confounds the association between education and hypertension, as it is associated with both the outcome (hypertension) and the variable of interest (education):

- Association between age group and hypertension: OR 1.27 (95% CI 1.26 to 1.28); and
- Association between age group and education: OR 0.93 (95% CI 0.92 to 0.94).

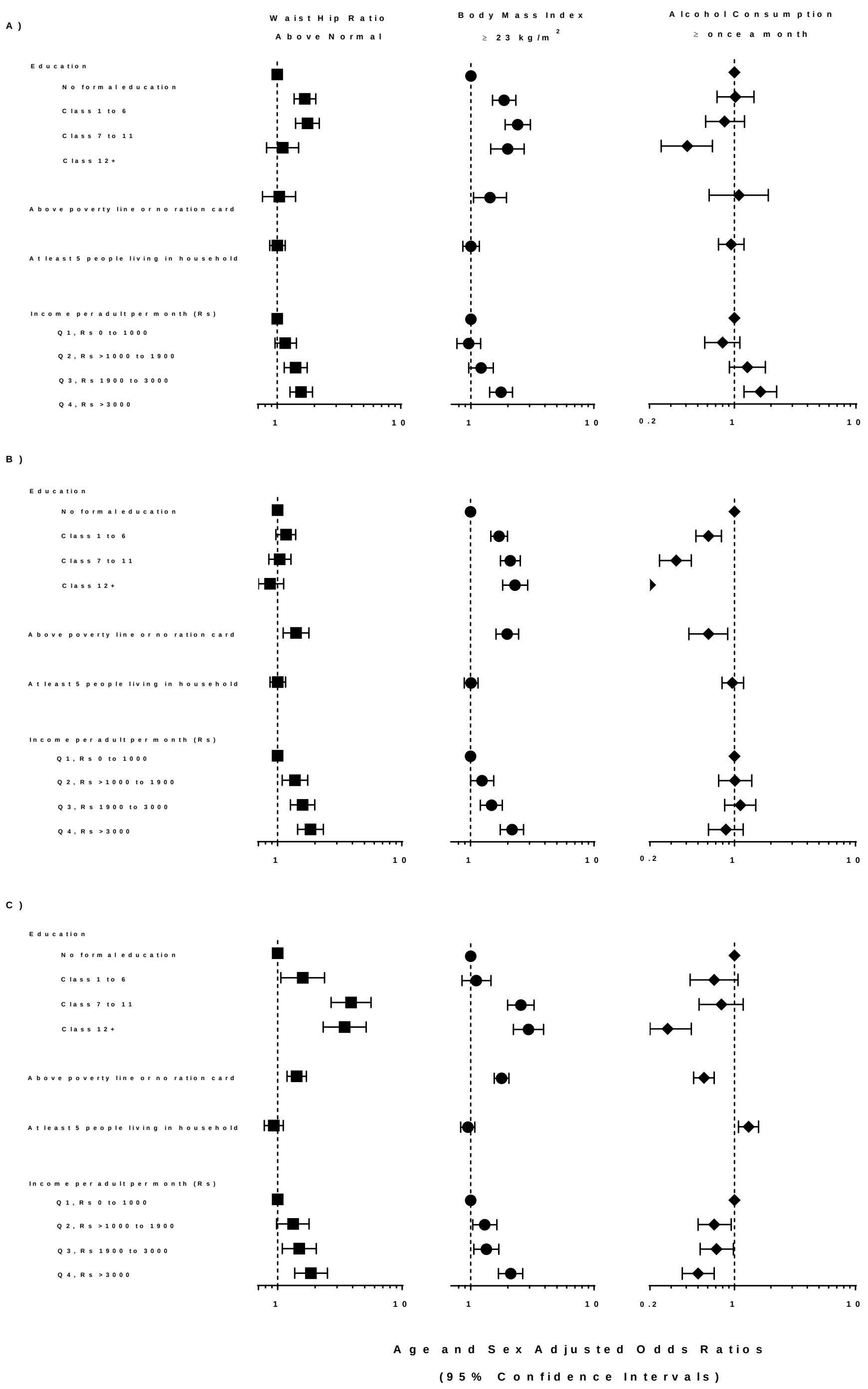

Figure S2. Association between Different Measures of SEP and Waist-Hip Ratio Above Normal, Body Mass Index ≥23kg/m^2 and Alcohol Consumption at least once a month in Three Rural Regions in India, 2014–2015: A) Rishi Valley; B) Godavari; and C) Trivandrum.

Abbreviations: Q1-4, quartiles 1-4; Rs, rupees

Error bars indicate 95% confidence intervals. Class 12+ includes individuals who graduated from secondary schooling, completed technical college or completed university. *P* values are the outcomes of logistic regression with hypertension as the dependent variable and the categorized terms for education (and income) introduced as a continuous independent variable.

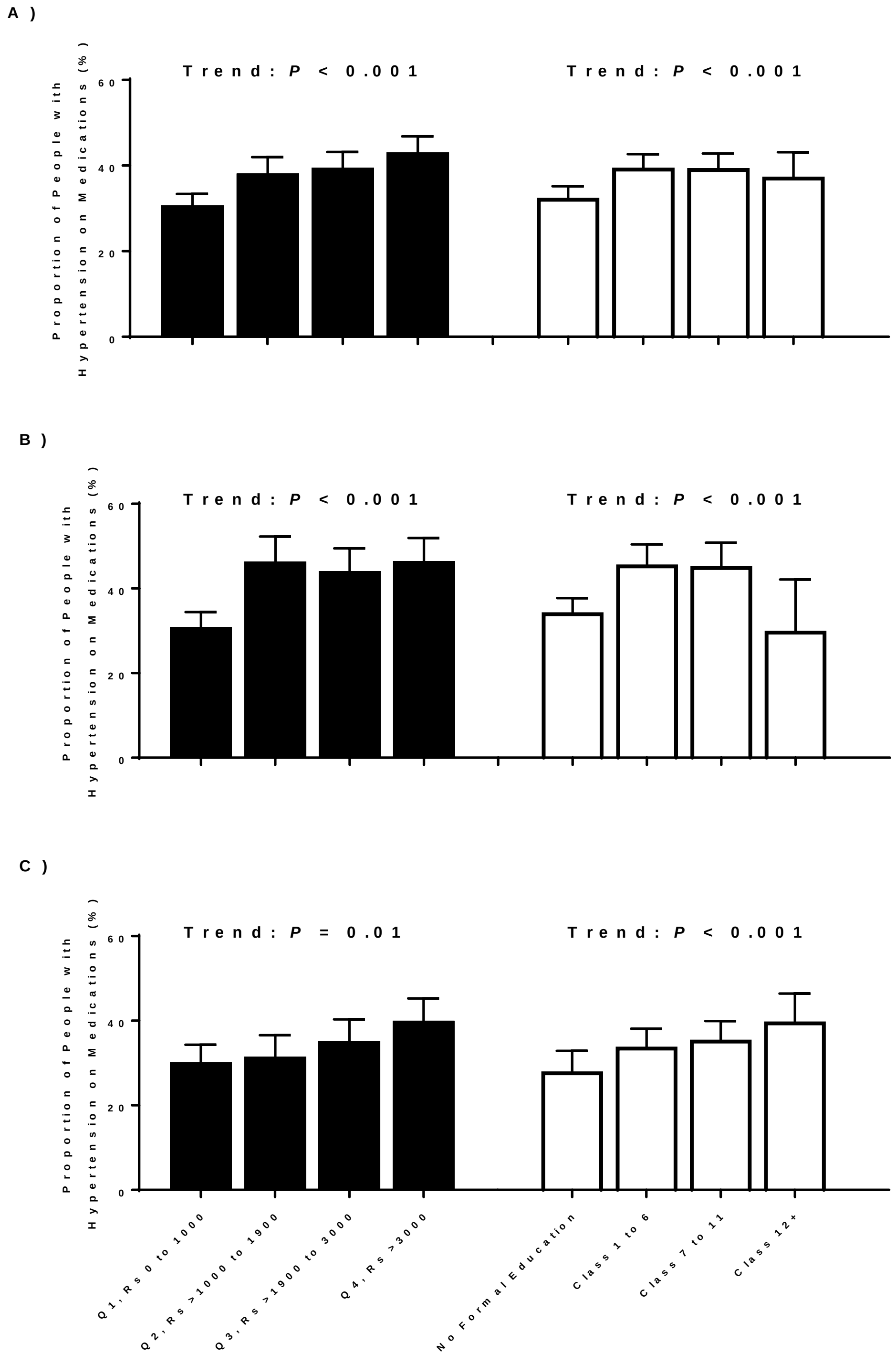

Figure S3. Proportion of people with hypertension reporting use of medications for hypertension in: A) women and men (n = 3,310); B) women (n = 1,687); and C) men (n = 1,618).

Abbreviations: Q1-4, quartiles 1-4; Rs, rupees

Error bars indicate 95% confidence intervals. Class 12+ includes individuals who graduated from secondary schooling, completed technical college or completed university. *P* values are the outcomes of logistic regression with hypertension as the dependent variable and the categorized terms for education (and income) introduced as a continuous independent variable.

Supplemental References:

1. State Planning Board, Government of Kerala. Human Development Report 2005. 2005. http://planningcommission.nic.in/plans/stateplan/sdr_pdf/shdr_kerala05.pdf (accessed 9 November, 2018)
2. Government of India. Census 2011. 2015. http://www.census2011.co.in/district.php (accessed 9 November, 2018)
3. Thrift AG, Evans RG, Kalyanram K, Kartik K, Fitzgerald SM, Srikanth V. Gender-specific effects of caste and salt on hypertension in poverty: a population-based study. *J. Hypertens.* 2011;29:443-450
4. Banerjee AV, Duflo E. The Economic Lives of the Poor. *J. Econ. Perspect.* 2007;21:141-167
5. Whelton PK, Carey RM, Aronow WS, Casey DE, Jr., Collins KJ, Dennison Himmelfarb C, DePalma SM, Gidding S, Jamerson KA, Jones DW, MacLaughlin EJ, Muntner P, Ovbiagele B, Smith SC, Jr., Spencer CC, Stafford RS, Taler SJ, Thomas RJ, Williams KA, Sr., Williamson JD, Wright JT, Jr. 2017 ACC/AHA/AAPA/ABC/ACPM/AGS/APhA/ASH/ASPC/NMA/PCNA Guideline for the Prevention, Detection, Evaluation, and Management of High Blood Pressure in Adults: Executive Summary: A Report of the American College of Cardiology/American Heart Association Task Force on Clinical Practice Guidelines. *Hypertension*. 2018;71:1269-1324
